# Biochar/Biopolymer Composites for Potential In Situ Groundwater Remediation

**DOI:** 10.3390/ma17163899

**Published:** 2024-08-06

**Authors:** Marco Petrangeli Papini, Sara Cerra, Damiano Feriaud, Ida Pettiti, Laura Lorini, Ilaria Fratoddi

**Affiliations:** 1 Department of Chemistry, Sapienza University of Rome, Piazzale Aldo Moro 5, 00185 Rome, Italy; marco.petrangelipapini@uniroma1.it (M.P.P.); ida.pettiti@uniroma1.it (I.P.); laura.lorini@uniroma1.it (L.L.); ilaria.fratoddi@uniroma1.it (I.F.); 2 Research Center for Applied Sciences to the Safeguard of Environment and Cultural Heritage (CIABC), Sapienza University of Rome, Piazzale Aldo Moro 5, 00185 Rome, Italy; 3 Research Center for Nanotechnology Applied to Engineering of Sapienza (CNIS), Sapienza University of Rome, Piazzale Aldo Moro 5, 00185 Rome, Italy

**Keywords:** colloidal biochar, biopolymers, chitosan, alginate, potato starch, carboxymethylcellulose, composite material, trichloroethylene (TCE), in situ remediation, groundwater remediation

## Abstract

This study explores the use of pine wood biochar (BC) waste gasified at 950 °C as fillers in polymer matrices to create BC@biopolymer composites with perspectives in groundwater remediation. Four biochar samples underwent different sieving and grinding processes and were extensively characterized via UV–Vis, FTIR, and FESEM–EDS, highlighting the fact that that BCs are essentially graphitic in nature with a sponge-like morphology. The grinding process influences the particle size, reducing the specific surface area by about 30% (evaluated by BET). The adsorption performances of raw BC were validated via an adsorption isotherm using trichloroethylene (TCE) as a model contaminant. A selected BC sample was used to produce hydrophilic, stable polymer composites with chitosan (CS), alginate (ALG), potato starch (PST), and sodium carboxymethylcellulose (CMC) via a simple blending approach. Pilot sedimentation tests over 7 days in water identified BC@PST and BC@CMC as the most stable suspensions due to a combination of both hydrogen bonds and physical entrapment, as studied by FTIR. BC@CMC showed optimal distribution and retention properties without clogging in breakthrough tests. The study concludes that biopolymer-based biochar composites with improved stability in aqueous environments hold significant promise for addressing various groundwater pollution challenges.

## 1. Introduction

In recent years, water pollution has been a pervasive issue worldwide and has posed a significant threat to both human health and the ecosystem [1,2]. Among different waterbodies and natural water resources, groundwater is one of the most important freshwater sources and is seriously threatened by anthropogenic activities, such as industrial and agricultural practices [3]. Concerning groundwater pollution, trichloroethylene (TCE) is one of the most hazardous organic contaminants, and it is widely used, from agriculture to industry and medicine [4]. Due to its chemical characteristics, i.e., stability under aerobic conditions, low flammability, low solubility in water, low viscosity, and higher density than water, TCEs show high persistence in the environment, being classified as contaminants of concern (COCs) in groundwater [5]. To date, lots of TCE groundwater remediation technologies have been developed both in situ (treatment of the contaminated matrix in its location) and ex situ (removal of the contaminated matrix and subsequent treatment in a different location) [6]. Depending on the mechanism underlying the trichloroethylene removal or degradation, they can be classified into three main categories: chemical (e.g., chemical oxidation using oxidants, such as KMnO_4_), physical (e.g., air stripping, pump-and-treat, adsorption), and bioremediation (e.g., biological reductive dechlorination of TCE) [7]. Besides common ex situ treatments, in situ technologies are potentially more sustainable from an economic and environmental standpoint [8]. Thus, in situ treatments represent preferable options, and, in this regard, absorption methods (physical capture) are gaining attention due to their operational simplicity, recyclability, high efficiency, and environmental friendliness [9]. Indeed, in in situ treatments, contaminants show high affinity for the adsorptive matrix, thus leading to little to no desorption/release into the groundwater environment after immobilization, achieving a long-term remediation [10]. Recently, new in situ adsorption processes have been developed based on injectable colloidal activated carbon (CAC) [11,12], zeolites [13,14], organoclays [15,16], organo-hydrotalcites [5], and graphene oxides [17]. However, their applications remain niche due to challenges in distributing the material effectively without clogging porous media. Proper suspension retention is not a simple task: too little retention may cause failure in achieving plume break, while too high retention may cause clogging issues that result in groundwater bypass [15].

As a cheap and eco-friendly material for this purpose, biochar (BC) is one of the most interesting alternatives to conventional absorbers. Biochar is a biomass-derived C-rich material obtained via pyrolysis or gasification of different feedstock, such as wood, agricultural waste, manure, and wastewater sludge. Biochar itself possesses a large specific surface area, high porosity, and thermal resistance, although its properties and sorption behavior depend not only on biomass feedstock but also on production temperature [18,19]. Depending on the biomass feedstock, different biochar chemical compositions and properties can be obtained. Wood-derived biochar shows a higher specific surface area and porosity due to the thermal stability of lignin and tends to have lower pH values than biochar obtained from agricultural and organic feedstock [20,21]. Agricultural- and organic-wastes-derived biochar show a lower cation exchange capacity [20,22]. Thus, biochar obtained from woody biomass is more suitable for organic pollution remediation and greenhouse gas emission reduction, while biochar with high ash content (derived from manure, sludge, etc.) are more suitable for cationic organic and heavy metal pollution removal. Pine-wood-derived biochar has been extensively studied, resulting in the best woody-type biochar for trichloroethylene removal from synthetic groundwater environments, such as among rice husks and Iron–Eupatorium Shrubs BC [23].

Several applicative studies demonstrated that raw biochar could sorb a wide range of contaminants, ranging from organic compounds (toxic dyes, polycyclic aromatic hydrocarbons (PAHs), polychlorinated biphenyls (PCBs)) to metal and metalloids both in their cationic or anionic form (U^6+^, Pb^2+^, Hg^2+^, Cd^2+^, AsO_4_^3−^, AsO_2_^−^, SeO_3_^2−^, SeO_4_^2−^, CrO_4_^2−^) [24,25,26,27]. Despite its absorption abilities, the main disadvantages of unmodified biochar are due to its hydrophobic nature and surface charge, which influence its retaining and adsorption capacity. There is usually a fraction of the particle size distribution of BC that is fine enough to be considered as a colloid (particles with diameters smaller than a few tens of micrometers) [28] and thus characterized by greater stability in water. Colloid retention by porous media is primarily controlled by straining processes [29], which occur when colloids are trapped by bottlenecks formed by collector particles [30]. A critical ratio between colloid diameter (d_p_) and collector diameter (d_c_) determines straining (these ratios vary between 0.027 [31] and 0.0017 [32]), although straining is also controlled by physicochemical factors, such as ionic strength and fluid dynamics; increasing velocity reduces straining intensity by destabilizing colloids attached to secondary minimum straining sites [33]. However, this fraction is a minority (1–20 mg·g^−1^) [34], and strategies to avoid rapid coalescence and aggregation of biochar in aqueous environment are needed.

In this framework, BC shows intriguing properties as a superior filler in polymer and biopolymer-based composites [35]. Concerning polymer matrices, BC has been used in several composite formulations in combination with synthetic and natural polymers with significant enhancement of either mechanical or electrical properties even at low concentrations [36]. Biopolymers such as polylactic acid (PLA), poly(3-hydroxybutyrate) (PHB), starch-based polymers, and biopolymers from algae biomass have the potential to be used as valuable flexible matrices to produce high-quality biochar composites and to establish a sustainable circular economy globally [36,37,38,39].

For example, T.-M. Nguyen et al. [40] studied biodegradable hydroxypropyl methylcellulose (HPMC), hydroxyethyl cellulose (HEC), and polyvinyl pyrrolidone (PVP) as binders for a persulfate/biochar barrier to treat trichloroethylene (TCE) in groundwater via an oxidative mechanism. A polyhydroxybutyrate (PHB)–biochar mixture was studied for trichloroethylene adsorption and removal [41]. Among others, chitosan/chitin-modified biochar composites were extensively tested for metal cations and dissolved organic matter (DOM) removal from wastewater, pointing out an improvement in the sequestration abilities and affinity for the contaminants (compared with pristine biochar) due to the presence of a polymer matrix [42,43,44]. The effect of a carboxymethylcellulose (CMC) and humic acid (HA) matrix on the stabilization of colloidal activated carbon (CAC) was studied, evidencing that CMC is able to provide a high mobility in water-saturated sediment, being fully available as adsorbent for organic contaminant [12].

Biopolymer-based BC composites still require optimization to reach performances comparable to traditional active carbon materials and take advantage of the synergistic effects of biochar dispersed in polymers. In this work, biochar powders (BCs) derived from pine wood gasification at 950 °C were used as an active carbonaceous material filler of biopolymer matrices such as chitosan (CS), alginate (ALG), potato starch (PST), and carboxymethylcellulose (CMC) to obtain BC@biopolymer composite materials.

The specific goals of this work were as follows: (i) synthesize biopolymer-based biochar composites to improve the aqueous stability of BC; (ii) give an insight into the chemical and structural characteristics of optimized BC@PST and BC@CMC composites. Emphasis was placed on the evaluation of the colloidal stability via spectroscopic techniques, with the aim to elucidate the role of polymers in stabilizing biochar matrix. In this context, only a few articles focus on the stability evaluation of biochar dispersed in biopolymers, revealing a possible interaction mechanism between counterparts (biochar and polymer). This information is of crucial importance to improving the distribution properties and adsorption of organic contaminants in practical applications. Indeed, the availability of an aqueous suspension of stabilized biochar allows us to perform TCE in an in situ remediation with injectable permeable reactive barriers (IPRBs) as an alternative to traditional PRBs. In situ injectable barriers allow us to achieve the plume break, interrupting the contaminants migration pathway, considering a combination of the single- and multi-layer physical adsorption processes [45]. The installation of IPRBs is operationally advantageous compared to the use of a PRB, as the volume of the excavation is reduced, and well drilling can be achieved at greater depths (>30 m in depth is a challenge) without high skill compared to trench excavation [46,47,48]. Although, the main challenges of operating IPRBs are related to reactive species distribution control, especially in highly heterogeneous aquifer matrices. In view of practical application purposes, the transport properties in water were evaluated. Preliminary column tests on TCE removal were conducted on raw biochar to assess adsorptive capacities. TCE was selected as the contaminant of choice due to its importance in contamination scenarios [49], and because it has been demonstrated (in previous studies) how the adsorption of TCE on BCs can promote the establishment of a beneficial biological reductive dechlorination process [50]. Compared with similar technologies, the herein-proposed modification of biochar allows us to obtain a stable aqueous suspension as a potential injectable permeable reactive barrier for in situ groundwater remediation, involving advantages related to (i) suspension stability, (ii) synthesis in mild conditions (room temperature or 70 °C), (iii) biodegradability, (iv) no toxic chemicals and complex equipment required, and (v) easy scalability. All materials were characterized via field-emission scanning electron microscopy (FE-SEM), N_2_ adsorption/desorption measurements, UV–Visible, and infrared (FT–IR) spectroscopy. The preparation of the herein-presented stable biopolymer-based biochar composites represents a promising low-cost, green, and effective sorbent in groundwater remediation strategies.

## 2. Materials and Methods

Biochar powders (BCs) derived from pine wood gasification at 950 °C were used as active carbonaceous material fillers of polymer matrices. Specifically, four different BC samples were studied based on different sieving and grinding processes, as described in Table 1. On the selected BC sample (AP sample), water-based BC@biopolymer composite materials were obtained by combining raw BC with commercially available biopolymers, such as chitosan (CS), alginate (ALG), potato starch (PST), and carboxymethylcellulose (CMC) as host matrices. Different concentrations of biochar (0.3–1 g·L^−1^) and biopolymers (0.2–20 g·L^−1^) were tested, and a one-pot blending mode at different temperatures (from room temperature up to 70 °C, depending on polymer solubility) was used as a simple and straightforward strategy to obtain BC@biopolymer composites. To improve the physicochemical properties of the potato-starch-based composite, an alkylpolyglucoside-based surfactant (APG2, a non-ionic surfactant) was added in a 0.1–5.0% *v*/*v* range. An APG-based surfactant was chosen according to its eco-friendliness, nontoxicity, and biodegradability [51]. The functional properties of optimized formulations were tested in breakthrough tests to assess transport properties, such as hydrodynamic dispersion and retention percentage, in various aquifer configurations. Batch adsorption isotherms on trichloroethylene (TCE) as a model contaminant were carried out to confirm the adsorptive capacity of the BC-based composites, which had already been validated in the literature [23].

### 2.1. Pine Wood Biochar Production

Pine wood biochar (BC) was used to produce the composite material (from European pine, Plößberg, Germany). The BC used is a waste product of a biomass energy production process implemented by Burkhardt Energy and Building Technology (Plößberg, Germany). The energy production involves the use of two machines in series: the first (V3.90) carries out the gasification of pine wood pellets at 950 °C, while the second (CHP ECO 220) produces thermal and electrical energy from the combustion of the previously produced syngas. The BC in question is, therefore, a by-product of the heat and power generation process that is usually disposed of as waste. Two replicated batches were used (A and B) and compared with the two samples obtained after the sieving and grinding procedure (AP and BP), respectively (see Table 1).

### 2.2. Preparation of BC@Biopolymer Composites

For the synthesis of the BC@biopolymer composites, biochar from pine wood and the following commercially available bio-based polymers (all Merck Sigma-Aldrich, Milan, Italy) were used: chitosan (CS), sodium alginate (ALG), potato starch (PST), and sodium carboxymethylcellulose (CMC). To optimize the composite formulation, the biochars, in the 0.3–1.0 g·L^−1^ range, were mixed with different concentration of biopolymers: 0.2, 5.0, 7.5, 10.0, 15.0, and 20.0 g·L^−1^. Pilot tests were conducted in a total volume of 10 mL with 0.30 g·L^−1^ BC concentration, whereas the final formulations were scaled-up to 1.5 L and 1 g·L^−1^ of BC. In a typical procedure, the selected amount of polymer powder was mixed with BC in a glass vial and the solid mixture mechanically homogenized with a spatula. A total of 10 mL of ultra-pure water (H_2_O_up_, 18.3 MΩ·cm, produced with a Zeneer Power I Scholar-UV instrument, Full Tech Instruments, Rome, Italy), was added, and the mixture was sonicated for 30 min. Then, to obtain the final composite, the aqueous suspension was vigorously stirred at different experimental conditions depending on polymer solubility, as reported in Table 2. In the case of the BC@PST composite obtained at 70 °C, it was allowed to cool down to room temperature, and 1.0% *v*/*v* of APG2 surfactant (Chimec, Italy) was added, whereas BC@CS, BC@ALG, and BC@CMC were synthesized in absence of further additives. The as-prepared final composites were used without further modification or purification. As a blank sample, a pristine BC aqueous suspension (denoted as BC) and BC + 1.0% *v*/*v* APG2 aqueous solution (denoted as BC/APG2) in the 0.2–5.0% *v*/*v* range were prepared.

### 2.3. Sedimentation Tests

To investigate the stability over time of the BC composites, sedimentation tests were performed as follows: the as-prepared BC@polymer composites were kept in a quiescent state for 7 days in a glass vial without stirring. For the UV–Vis and DLS analysis, a sample aliquot (1 mL) was taken from 1/3 of the total volume and diluted 1:3 *v*/*v* with H_2_O_up_. For the test, the biochar concentration was fixed at 0.3 g·L^−1^, and the absorbance values at 650 nm at 0 h, 24 h, 7 days (Abs_t_) were measured. The sedimentation percentage of BC inside the polymer matrix at different times was calculated using Equation (1) [52].

Sedimentation (%) = (1 − (Abs_t_/Abs_t0_))·100 (1)

### 2.4. Adsorption Isotherms

Batch adsorption tests were carried out with TCE to verify the efficiency of the raw BC in immobilizing the chosen contaminant in an aquifer medium. The BC used in these tests was subjected to the same treatment as that used in the distribution tests (Section 2.5), i.e., sieving at 64 µm and manual grinding. Five different loads of BC were placed in the borosilicate glass reactors: 10, 20, 30, 40, and 50 mg. For TCE, a 25 mg·L^−1^ water solution was prepared in a 1 L Tedlar bag (Supelco, Bellefonte, PA, USA) to avoid headspace formation, adding a volume of 17 µL of pure TCE (ACS ≥ 99.5%, Sigma-Aldrich, St. Louis, MO, USA). To verify the initial effective TCE concentration, the contaminated solution was sampled and analyzed before setting up the tests. The solution was horizontally shaken with an agitator shaker (ASAL Universal Table Shaker 709) for three days to reach complete solubilization of TCE. The batch reactors (VWR International glass vials, Milan, Italy) were prepared by weighing a known amount of BC and fully filling them with approximately 0.02 L of the contaminated solution. The reactor was sealed by a Teflon butyl stopper (Wheaton, Millville, NJ, USA) and an aluminum cap and mechanically shaken with a vertical rotating mixer (Biologix MX-RD-E, Camarillo, CA, USA) for 24 h. At the end, the samples were left to sediment, and an aliquot of the liquid phase was withdrawn with a syringe and analyzed. Each load of BC was performed in triplicate to strengthen the data.

The Freundlich constants K_F_ and n were determined using SigmaPlot 12.0 software and fitted with a Freundlich-type isotherm (Q_e_, Equation (2)) [53] as it gave an optimal representation of the trend.

Q_e_ = K_F_C_eq_^n^ (2)

The equilibrium concentration C_eq_ in the liquid was carried out via a gas chromatographic analysis sampling the contaminated solution. The determination of the concentration of TCE adsorbed on the BC (defined as S, mg_TCE_·g_BC_^−1^) is calculated using Equation (3) [54]:

S = (C_0_V − C_eq_V)/m (3)

where C_0_ is the initial TCE concentration in the contaminated solution; V and m are the volume of solution and the mass of BC loaded in each reactor, respectively.

### 2.5. Continuous Flow Column Distribution Tests

Column transport tests were performed to verify the deliverability of the produced composites and their retention in the simulated aquifer. The columns used in these tests are made of PMMA and have dimensions of 14 × 2.5 cm. The columns were packed with 600–800 µm diameter glass beads to represent an aquifer consisting of medium sand. The columns were equipped with two sampling points: one for the inlet and one for the outlet.

The experimental setup comprises the following steps: (1) continuous feeding of the analyzed solution (tracer and BC@polymer composite suspension) taken from a magnetically stirred beaker via a peristaltic pump (Gilson miniplus evolution) and then injected into the column in an up-flow configuration, to avoid the formation of air pockets; (2) the solution exiting the top of the column was collected in glass tubes by a Gilson Fraction Collector 201-202 that was used to monitor the effluent leaving the column. The concentration of BC and BC-based composites in the effluent was monitored by measuring the turbidity of the suspension with a photometer at 650 nm. A calibration curve was performed for each test with a different batch of BC composite to ensure accurate quantification. The tests were carried out in two phases marked by a change in feed: (i) distribution of the BC suspension within the column and (ii) washing with water to verify the amount of BC retained by the column, and thus closing the mass balance.

The amount of BC retained in the column was determined by the mass balance in Equation (4).

m = Σ_i_^n^(Q_i_C_0_ − Q_i_C_i_) (4)

where Q_i_ is the flow rate at i-th time (although designed as constant flow tests some fluctuated significantly), C_0_ is the concentration of BC entering the column, C_i_ is the concentration of BC leaving the column at i-th time, and m is the BC mass retained by the column. Prior to the BC distribution tests, a tracer test with 150 mg·L^−1^ of Cl^−^ (supplied as NaCl) feeding solution was carried out to determine pore volume (PV), effective porosity (ε), and hydraulic retention time (θ). The effluent samples obtained by the aforementioned fraction collector were subsequently analyzed with an ion chromatograph in the case of tracer tests and with a UV photometer at 650 nm in the case of BC distribution tests. Both the tracer and distribution tests were carried out with flow rates of approximately 0.6 mL·min^−1^ and apparent velocity of 0.3 cm·min^−1^. The results of the continuous tests were plotted, indicating the ratio of outlet analyte concentration (C) over inlet analyte concentration (C_0_) on the ordinates (C/C_0_) and the ratio of fed eluent volume over effective column volume (Pore Volumes or PVs).

### 2.6. Characterization Techniques and Analytical Methods

Absorption spectra were recorded using a UV–Visible spectrophotometer Varian Cary100 instrument (Agilent Technologies, Milan, Italy) in a 200–800 nm wavelength region. Quartz cuvettes with a path length of 1 cm were used in all experiments.

Turbidity measurements on column effluent for BC composites distribution tests were carried out with a UV–Vis-NIR Shimadzu UV1800 photometer at 650 nm, with 1 cm cuvettes in polystyrene.

pH measurements were carried out using pH600 Eutech Instruments pHmeter (Eutech Instruments Pte, Singapore) calibrated with standard solutions (pH 4–10) before measurement.

Fourier transform infrared (FT–IR) spectroscopy in Attenuated Total Reflectance (ATR) mode were performed using a Bruker Vertex70 instrument (Bruker, Milan, Italy) over the wavenumber range of 4000–600 cm^−1^ with a resolution of 4 cm^−1^ and 32 scans. The samples were deposited as a solid directly onto the diamond-coated ATR crystal.

The specific surface area (Brunauer–Emmett–Teller (BET) method in the 0–0.1 p/p° interval) [55], total pore volume, micro-pore volume, and pore size distribution were determined by adsorption/desorption of N_2_ at −196 °C (77 K) using a 3Flex 3500 Micromeritics analyzer (Micromeritics, Norcross, GA, USA). A total of 0.250 g of the powder samples were pretreated at 250 °C for 24 h in an oven (ambient pressure) to remove excess of absorbed water (calculated as weight loss %), at 300 °C for 2 h, and at 350 °C for 1 h under vacuum via thermally controlled heating mantles, up to a residual pressure lower than 0.5 Pa. The pore size distribution was determined using the Barrett–Joyner–Halenda (BJH) method [56] from the adsorption isotherm. The total pore volume was obtained using the rule of Gurvitsch [57]. The micro-pore volume was obtained via the t-plot. The uncertainty was ±5 m^2^·g^−1^ for the specific surface area values and ±0.005 cm^3^·g^−1^ for the total pore volume values.

The size as hydrodynamic diameter (<2R_H_>), size distribution (PDI), and ζ-potential were evaluated by dynamic light scattering (DLS) using a Malvern Nano-ZetaSizer apparatus, operating in backscattering mode (173°) and equipped with a 5 mW HeNe laser (λ = 632.8 nm). Both the size and ζ-potential were measured at 25 °C using a minimum of ten replicates and presented as mean value ± standard deviation of the data.

Surface morphology of the materials was investigated by field-emission scanning electron microscopy equipped with an energy-dispersive X-ray detector (FESEM–EDS) on an Auriga Zeiss instrument (ZEISS Microscopy, Jena, Germany). The samples were drop-casted onto a silicon stub from their aqueous suspension without any conductive coating and air-dried. The acceleration voltage was set at 1.5 kV. The FESEM images were analyzed using ImageJ software 1.54j.

A chloride analysis for the tracer tests was performed with a Dionex ICS-1000 IC ion chromatograph (Waltham, MA, USA) equipped with an electrical conductivity detector and Dionex AS-40 autosampler (Waltham, MA, USA)). The instrument is equipped with a Dionex IonPac AG14 pre-column (4 × 50 mm), a Dionex IonPac AS14 IC column, and a Thermo Fisher Scientific AESR 500 4 mm suppressor. The eluent phase was prepared with 3.5 mM of Na_2_CO_3_ and 1.0 mM of NaHCO_3_, with 1.2 mL·min^−1^ as the flow rate. The chloride calibration curve was realized from 5 to 160 mg·L^−1^ of Cl^−^ (supplied as NaCl).

TCE concentration for isothermal curve determination was carried out with a DANI MASTER GC (DANI Instruments, Contone, Switzerland) gas chromatograph, equipped with DANI 86.50 headspace auto-sampler, TRB624 capillary column (30 m × 0.53 mm ID × 3 µm), and a Flame Ionization Detector (FID). The DANI 86.50 was set up as follows: oven temperature 80 °C, manifold temperature 120 °C, transfer line temperature 180 °C, shaking softly for 1 min. The GC conditions were as follows: N_2_ carrier gas (flow 10 mL·min^−1^), 180 °C injector temperature split injection 1:2; 200 °C detector temperature with air, N_2_ and H_2_ for the FID (flows 240, 25, 60 mL min^−1^). The oven temperature was programmed as follows: 60 °C for 3 min, 30 min to 120 °C, then 6 min at 120 °C. For the quantitative determination of TCE, a calibration curve was obtained by dilution of a TCE/ethanol stock solution in standards with a concentration range of 0.1–25 mg·L^−1^.

### 2.7. Statistical Analysis

Experiments were conducted at least in triplicate, and the data were analyzed using SigmaPlot 12.0 and OriginPro 8.0 software. All data were reported as mean value ± standard deviation. For calibration curves, linear regression was used to fit the data and to find the best equation from which fitting parameters (intercept, slope) were extrapolated.

## 3. Results and Discussion

Herein, the pine wood biochars obtained via gasification at 950 °C were used as the starting material to obtain composites with different biopolymers. The aim is to obtain a cheap, colloidally stable, and environmentally friendly material with application as an adsorbent towards chlorinated hydrocarbons. In this regard, biochars produced at high temperatures are reported to be more effective than those produced at lower temperatures (200–600 °C), mainly due to their improved capacity to act as an organic compound sequester, higher surface area, and nano-porosity [58]. In the following section, extensive characterizations of both raw biochar and composite materials are presented, together with preliminary transport tests on optimized formulations. Prior to composite synthesis, raw BC adsorption properties were evaluated using TCE as a model contaminant.

### 3.1. Raw Pine Wood Biochar Characterization

Prior to application in composites, pine wood biochars (BCs) were characterized as raw materials. Four different BC samples (replicates of two different batches) obtained at 950 °C gasification temperature were studied, based on different sieving and grinding processes: A, B (sieving at 0.5 mm) and AP, BP (sieving 64 µm and manual grinding), see Table 1.

#### 3.1.1. Fourier Transform Infrared Spectroscopy (FTIR) and Morphological Analysis

**Figure 1:**
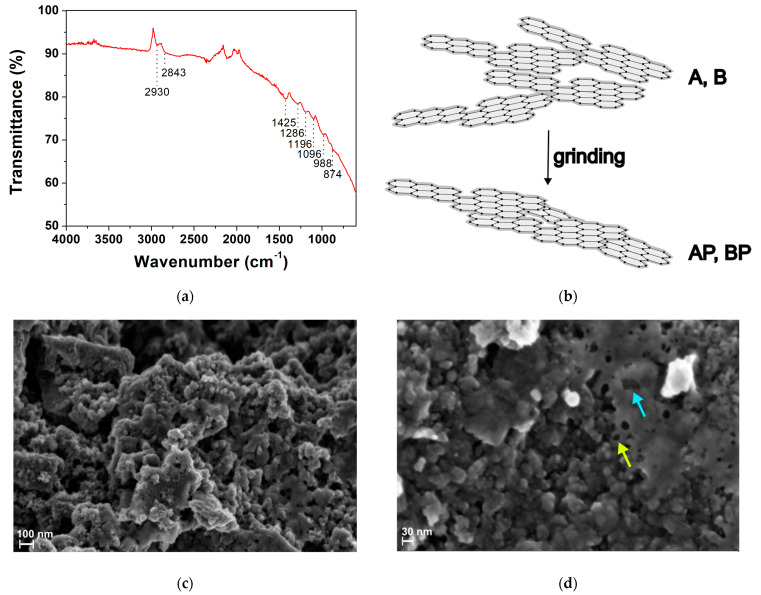
(**a**) The FTIR–ATR spectrum of raw pine wood biochar deposited as a solid powder. The spectrum of the AP sample is reported as a representative sample; (**b**) the graphitic structure of BC according to its structural features; (**c**,**d**) the representative field-emission scanning electron microscopy (FE-SEM) images of raw pine wood biochar (AP sample) at different magnification. The light blue arrow indicates a typical large pore; the yellow arrow denotes a typical small pore on BC surface. The sample was drop-casted onto a silicon stub from its aqueous suspension. The accelerating voltage was 1.50 kV.

Infrared spectroscopy was used to assess the chemical surface structure and the presence of functional groups. Since the gasification temperature (950 °C for all samples) determines the content of surface functionalities, a representative FTIR spectrum in the ATR mode of the AP sample is presented (Figure 1a). The bands between 2950 cm^−1^ and 2850 cm^−1^ were associated with the asymmetric and symmetric C–H stretching vibrations (ν_as_, ν_s_) of aliphatic carbons ν_as_(–CH_2_) = 2930 cm^−1^ and ν_s_(–CH_2_) = 2843 cm^−1^, which was also confirmed by the –CH_2_ scissoring vibration (partially overlapped with C–C stretching of aromatic ring) at 1425 cm^−1^ and 2890 cm^−1^ –CH– stretching. The bands at 1286 cm^−1^, 1096 cm^−1^, and 1196 cm^−1^ correspond to C–O and C–O–C stretching, attributable to the presence cellulose, hemicellulose, and lignin residual fractions in the biochar structure [59]. The weak bands between 988 and 874 cm^−1^ can be attributed to aromatic C–H bending vibrations, suggesting the presence of aromatic hydrogens in the biochar structure, as expected for lignin-based BC. No presence of typical hydroxyl group stretching vibration (ca. 3200 cm^−1^), as well as carboxyl C=O stretching (ca. 1730 cm^−1^), was detected due to dehydration reactions; although, a small quantity of oxygen-containing organic groups was still retained even during pyrolysis or gasification at elevated temperature [60,61,62]. The signal at 2106 cm^−1^ was from instrumental background. The limited presence of functional groups and aromatic portion in the spectrum implied that BCs are essentially graphitic in nature (Figure 1b). Indeed, at the used gasification temperature, the aromatic structures condense, forming highly disordered graphite-like domains with less residual functional groups. The absence of specific absorption arose from the UV–Vis spectra of all biochars (Figure S1). Due to their chemical features, these materials are commonly classified as hard carbon due to their high mechanical hardness [38].

Morphologically, the raw AP biochar was examined via scanning electron microscopy. The images presented in Figure 1c,d showed a sponge-like topography of the surface, with a grain size of (37 ± 8) nm, see also Figure S2. The pores were quasi-spherical with an average pore size (taken from a total of ten SEM images) from (19 ± 6) nm to (123 ± 59) nm, depending on the pore and measurement orientation. The fluffier and inhomogeneous structure of the biochar agrees with a high pyrolysis/gasification temperature, which reduces the high percentage of cellulose, hemicellulose, and lignin in the biomass [63]. The elemental analysis was carried out by EDS (Figure S2). The biochar is mainly composed of carbon (>70%), oxygen (approx. 15%), and inorganic minerals (Na, Mg, Al, Si, S, P, Cl, Ca, K, Mn).

#### 3.1.2. Brunauer–Emmett–Teller (BET) Surface Area and Textural Parameters

The specific surface area and textural parameters (total pore volume and micro-pore volume) influence the adsorption performances of biochar and play a role in considering BC as a filler in the polymer matrices. High surface areas and pore volumes are reported to improve mechanical properties of BC@polymer materials, due to infiltration of the polymer matrix during the mixing process [64]. Prior to analysis, the samples were treated at 250 °C for 24 h in a conventional oven to remove excess of absorbed water, and the weight loss % (loss of moisture content) was calculated using the following Equation (5) [65]:

Weight loss (%) = (w_i_ − w_f_/w_i_)·100 (5)

where w_i_ is the initial weight of the biochar; w_f_ is the weight of biochar after drying process in the oven. The results of the weight loss are reported in Table 3.

**Figure 2:**
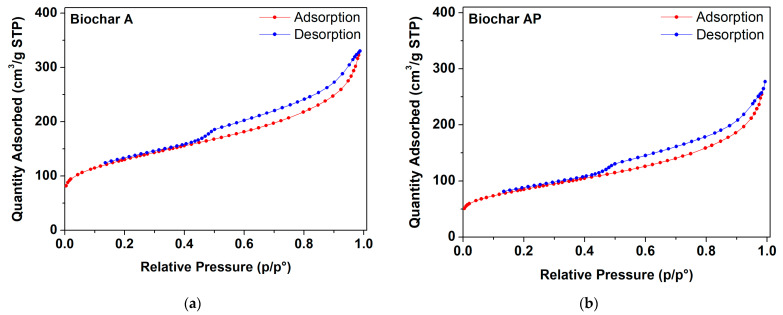

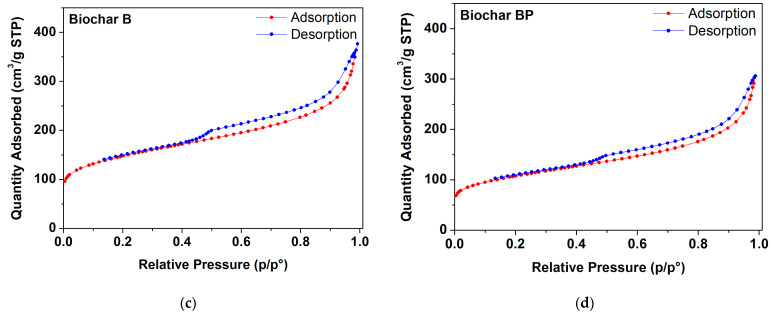
N_2_ adsorption/desorption isotherm for different raw biochar samples obtained at 950 °C: (**a**,**c**) sample A, B (sieving at 0.5 mm); (**b**,**d**) sample AP, BP (sieving at 64 µm and grinding).

The N_2_ adsorption/desorption isotherms of different biochar samples (A, AP, B, BP) subjected to different sieving and manual grinding procedures are reported in Figure 2. The curves are consistent with a type IV isotherm and type H4 hysteresis loop [66]. The results of N_2_ adsorption/desorption measurements are given in Table 3. All raw BC samples resulted to be micro-/mesoporous materials with a continuous pore size distribution in the 0–40 nm range (with the maximum pore size distribution at <1.5 nm). The pore size distributions are given in Figure S3. It is worth noting that a reduction in the specific surface area (m^2^·g^−1^) occurs in the samples after manual grinding (AP and BP samples). The grinding process (e.g., impact or shear) has been reported to alter the pristine molecular structure of the graphite-based samples, and due to weak intralayer bonds, additional structural distortions (besides pre-existing dislocations and stacking faults), such as rotation, translation, curvature, and fluctuation of interlayer spacing of graphene layers, can be easily induced [67]. Particularly, an increase in the specific surface area is accompanied by an increase in the interplanar distance of graphite layers. However, a suppression of the surface area (i.e., reduction in interplanar distances) can be induced through mild manual grinding (with mortar and pestle), in which shock (fracturing) forces predominates over shear (abrasive) forces, forming aggregates [68,69].

#### 3.1.3. Hydrodynamic Particle Size Distribution

**Figure 3:**
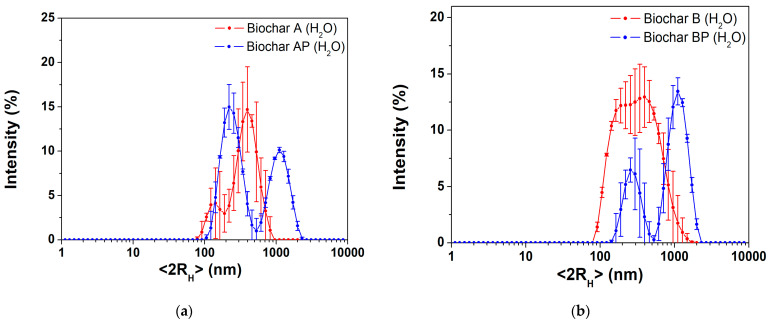
DLS size distributions in H_2_O of freshly prepared BC samples: (**a**) overlap A, AP; (**b**) overlap B, BP.

The mean particle size, evaluated as hydrodynamic diameter (<2R_H_>), of the raw biochars were evaluated via a dynamic light-scattering technique in water. The results of freshly prepared biochar suspension are presented in Figure 3. Biochar A (Figure 3a, red line) showed a population (90% of the particles) centered at (425 ± 98) nm, whereas a less intense population appears at (138 ± 34) nm. Manual grinding (sample AP, Figure 3a, blue line) slightly improves the intensity percentage of the smaller population centered at (247 ± 7) nm; although, aggregates were found above 1000 nm. Biochar B resulted in a broad distribution centered at (455 ± 174) nm (Figure 3b, red line), whereas in the BP sample two, population can be distinguished, the first appearing at (271 ± 46) nm and a second one at ca. 1000 nm (Figure 3b, blue line). The presence of a large population in the AP and BP samples further confirms the formation of larger aggregates after the manual grinding process (fracturing-type process) with a decrease in the interplanar distance of graphite layers constituting the biochar samples, as per specific surface area results (Section 3.1.2). Despite their hydrophobic nature, BCs showed a ζ-potential in the −19 to −30 mV range, at their native pH of 9 (determined in a 2:1 *w*/*v* of solid biochar to water). The results were as follows: A = (−31.9 ± 0.9) mV, AP = (−24.7 ± 0.8) mV, B = (−25.3 ± 0.9) mV, BP = (−19.1 ± 0.9) mV. After two hours of static aging, all BCs water suspensions showed an increase in the hydrodynamic diameter with a single population above 1500 nm (Figure S4) and a decrease in the ζ-potential value (A = (−13.9 ± 0.7) mV, AP = (−15.9 ± 0.4) mV, B = (−17.2 ± 02) mV, BP = (−5 ± 1) mV, Figure S4), highlighting the high-temperature biochar samples’ poor stability in aqueous environments.

Based on the above characterization results, it can be stated that gasification at 950 °C produces biochars with reproducible features, for sample A and B. The manual grinding process increases the intensity distribution of the smaller population (although showing a higher amount of aggregates compared with untreated samples), with the AP sample showing the higher percentage of colloidal fraction, i.e., it showed the most intense population below 200 nm, with respect to other BC samples. Moreover, the results showed that raw biochar possesses limited stability as aqueous suspension, with fast aggregation, due to the absence of specific surface functional groups. Due to reproducibility of the process, the AP biochar sample was selected as a reference material for further studies, as reported in the following section.

### 3.2. Adsorption Isotherm Curve of Trichloroethylene (TCE) of Raw Biochar

**Figure 4:**
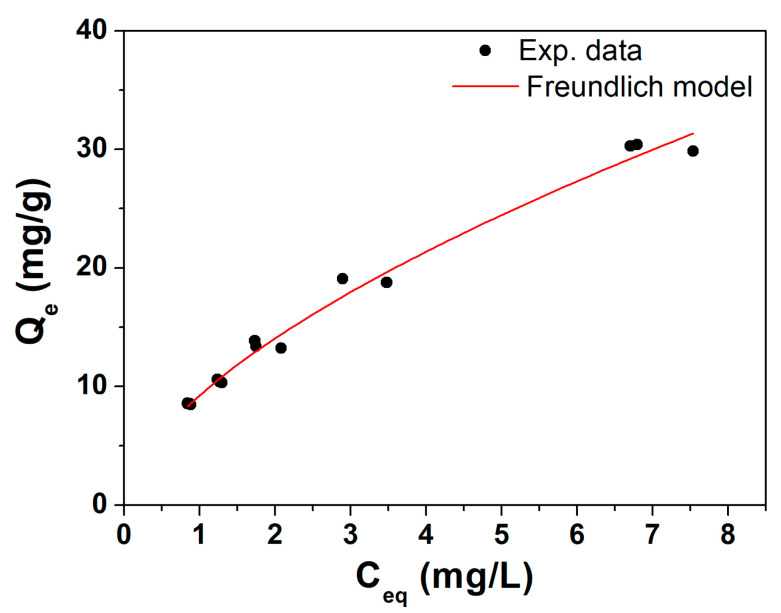
Isotherm of TCE adsorption on BC with Freundlich fitting equation. AP sample was taken as representative for this study.

Before the bio-composite synthesis, the raw AP sample was studied as an effective adsorber towards TCE as a contaminant of choice. In Figure 4, the isotherm of TCE adsorption obtained from the AP biochar sample is reported. As can be seen, experimental data follow a classic adsorption pattern of a solute on a solid adsorbent. In this case, one is working at relatively low ratios of mass of contaminant to the mass of adsorbent, thus staying well below the saturation threshold of the raw BC. The trend was confirmed by the curve pattern well described by a Freundlich isotherm, rather than a Langmuir-type one, models which fit well with the experimental test data (high R^2^ of Freundlich model). The Freundlich curve is suitable for heterogeneous surfaces with non-uniform distribution of the active site and was previously successfully applied for pine wood biochar [23].

Freundlich’s characteristic parameters obtained from the fit were K_F_ = 9.2 ± 0.3 (Lg^−1^) and n = 0.61 ± 0.02, indicating very good adsorption capacities for TCE compared to the literature values [23]. This preliminary evaluation validates the use of AP biochar (sieving at 64 µm and grinding) as an inorganic filler for subsequent polymeric composites and related column distribution tests.

### 3.3. BC@Biopolymer Composites Characterization

**Figure 5:**
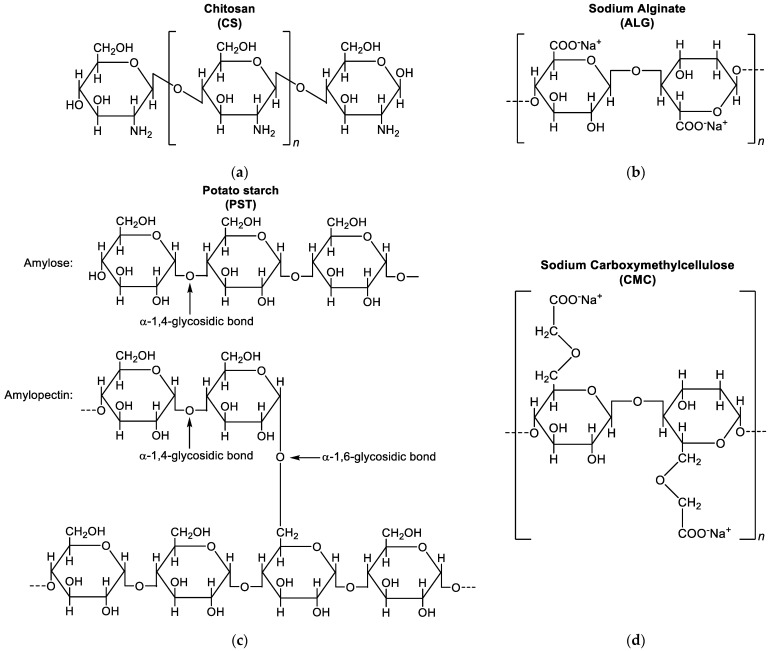
Molecular structure of naturally available polymers used for BC composites: (**a**) chitosan (CS); (**b**) sodium alginate (ALG); (**c**) potato starch (PST); (**d**) sodium carboxymethylcellulose (CMC).

The biopolymer-based biochar composite materials (obtained with the AP biochar sample) were prepared via a simple and straightforward blending approach at different temperatures, according to the chemical nature of polymers (see paragraph Table 2), using biochar derived from pine wood (950 °C gasification temperature). Indeed, raw biochar suffers from a lack of stability in water and to overcome these limitations, emphasis was put on the modification of biochar with different polymers in order to enhance its surface functionality (with positive impact on water stability) and pore structures. The polymers used in this study are reported in Figure 5. To do so, we evaluated the sedimentation and hydrodynamic parameters (size, size distribution, and ζ-potential) changes over time.

#### 3.3.1. Sedimentation Tests

**Figure 6:**
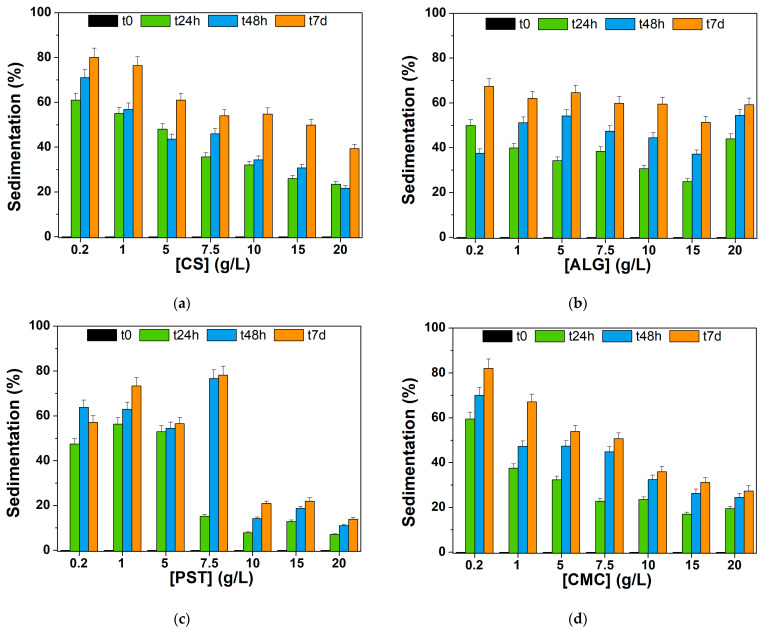
Dispersion stability over time evaluated as sedimentation percentage for 0.3 g/L of BC blended with: (**a**) chitosan (CS); (**b**) sodium alginate (ALG); (**c**) potato starch (PST) + APG2 1.0% *v*/*v*; and (**d**) sodium carboxymethylcellulose (CMC) at different concentrations.

To evaluate the stability in water over time of the biopolymer-based composites compared to the pristine biochar (stability < 2 h in water, see Section 3.1.3), preliminary static sedimentation tests (i.e., the quantity of the dispersed biochar settles down without stirring the solution) were conducted on different BC@polymer formulations, fixing the concentration of biochar at 0.3 g/L and optimizing the polymer concentration in the 0.2–20 g/L range. The sedimentation was calculated, monitoring the absorbance value at 650 nm for each formulation (after 1:3 *v*/*v* with H_2_O) at different time points (0, 24 h, 7 days), using Equation (1) reported in Section 2.3. As can be seen from Figure 6, a polymer concentration below 10 g/L resulted in an unstable dispersion (≥50% sedimentation) over a week in all formulations studied herein.

In the case of chitosan (CS, Figure 6a) and sodium alginate (ALG, Figure 6b), formulations at higher polymer concentration (>10.0 g·L^−1^) sedimented rapidly after 24 h, reaching (27 ± 5)% (on average) and (32 ± 10)% (on average), respectively. In this regard, sedimentation of the BC@ALG composites settled at around 60% after 7 days, demonstrating the feeble steadiness of the BC@ALG composites. Compared to the ALG, the BC@CS composites showed an improved stability within a week (39 ± 2%); although, chitosan showed the best solubility in acidic environments via primary amine protonation [70]. Indeed, the less stable formulation, i.e., BC@CS 0.2 g·L^−1^ showed a pH value of 6.63, whereas a higher CS concentration in the composite resulted in an acidic pH in the 4–5 range (see Table S1 for complete pH values). The poor solubility of the CS matrix at neutral and basic pHs strongly limits its applicability in environmental applications. Indeed, in the literature, chitosan is often used in form of chitosan–oxalate gel beads (obtained with addition of ammonia) [42], 2% acetic acid solution/NaOH [43], requiring purification steps before application.

The potato-starch-based composites (Figure 6c) showed a higher stability in the 10–20 g/L concentration range, in the presence of APG2 surfactant as additive in the mixture. Some important points should be highlighted when considering the BC@PST composites. First, potato starch is a densely packed biopolymer which consists of a mixture of two polysaccharides: amylose (20 to 35%) and amylopectin. Heating starch (below 100 °C) in excess water causes swelling of PST granules and loss of crystallinity in a semi-cooperative process. The subsequent cooling results in a transition from a liquid system with dispersed granules to an elastic gel (referred to as sol–gel transition), and for longer storage periods at room temperature, a reordering of the amylopectin chains also occurs with an increase in the elastic modulus of starch [71]. As a first attempt, the viscous gel-like structure formed after retrogradation can be responsible for a physical encapsulation of the BC granules. Indeed, potato-starch-based systems have been used over the years to physically entrap hydrophobic molecules (e.g., food ingredients, aroma compounds, etc.) [72]. However, the main drawback of PST-based composites is the high intrinsic viscosity, ca. 10^3^ times higher than the viscosity of water (expressed in cP) [73]. Thus, a slight improvement in the viscosity and stability of the BC@PST composites was achieved via the addition of a APG2 biodegradable surfactant (compared with composites without APG2 addition). APG2 allowed us to improve the wetting properties and water tolerance of the composite in a wide range of pHs [74]. The appropriate amount of surfactant was visually chosen upon stability tests within 7 days in the presence of 0.3 g/L of raw biochar, and the results are presented in Figure S5. Evaluating ten different APG2 concentrations (0.1, 0.2, 0.4, 0.6, 0.8, 1.0, 1.2, 1.4, 2.0, 5.0% *v*/*v*) above its critical micelle concentration (0.0071 wt% for APG2 [42]), the highest stability was shown by 1% *v*/*v* dispersion, thus being selected for the BC@PST composite’s preparation. The properties of the PST and APG2 combination allowed us to obtain stable composites, in which sedimentation is far below 30% (14 ± 1%) within 7 days of storage.

Considering the use of sodium carboxymethylcellulose as a polymer matrix for BC dispersion, results in Figure 6d showed that sedimentation percentage settled around 30% after a week: (36 ± 2)%, (31 ± 2)%, and (27 ± 2)% for 10.0, 15.0, and 20.0 g/L, respectively. Being an anionic polysaccharide, CMC shows characteristic coagulation and tends to form micellar-like structure absorbing a considerable amount of water [75], thus being suitable as a support matrix for biochar. Moreover, CMC shows some advantages compared with other polymers: (i) It does not require heating for complete dissolution; (ii) it is soluble in water in a wide range of pHs; and (iii) led to a slightly basic suspension (ca. pH 9, see Table S1 for complete pH values) with long-term stability. According to these results, the most stable and versatile composites (considering also the viscosity of the final suspension) resulted to be BC@PST + APG2 1.0% *v*/*v* (Figure 6c) and BC@CMC (Figure 6d); although, in the latter case, ca. 50% of the dispersed biochar sedimented after 7 days.

#### 3.3.2. Hydrodynamic Parameters of BC@PST and BC@CMC Composites

**Figure 7:**
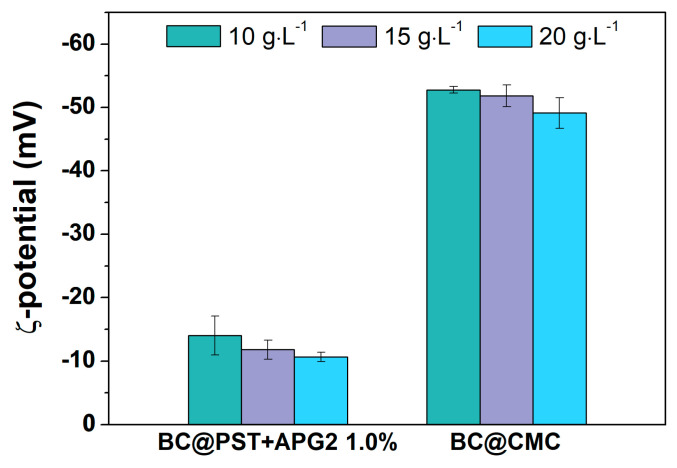
ζ-potential values for BC@PST + APG2 1.0% *v*/*v* and BC@CMC after 7 days at 10.0 g/L (green bar), 15.0 g/L (violet bar), and 20.0 g/L (light blue bar). Complete values can be found in Table S1.

The hydrodynamic diameter (<2R_H_>) and ζ-potential were recorded at different time points to further evaluate the stability and hydrodynamic behavior of the BC@polymer formulations. According to the sedimentation tests, the ζ-potential values of the selected BC@PST + APG2 1.0% and BC@CMC composites after 7 days of aging are reported in Figure 7 and discussed in the following section, whereas the full data (0 h, 24 h, 7 days for all composites) can be found in Table S1.

In the case of potato-starch-based composites, the ζ-potential value settled around (−14 ± 3) mV, (−12 ± 2) mV, and (−10.7 ± 0.8) mV, for 10.0, 15.0, and 20.0 g/L, respectively, with a decrease of about 15% over a week. The values for BC@CMC fluctuates around −55 mV at all time points, higher than that of similar polymer/BC composites [76]. Although BC@PST + APG2 formulations showed the lower sedimentation percentage compared to BC@CMC, the latter showed the highest colloidal stability over time. It is noteworthy that the ζ-potential value in polymers depends on the surface properties and size of granules, thus being a strong pH-dependent parameter [77]. Specifically, starch, both in its native and pasted form, is a very complex dispersion that cannot be clearly classified as a solution, colloid or suspension, and its hydrodynamic properties are affected by many factors, e.g., pH and presence of dispersed phases [66].

#### 3.3.3. FT–IR Characterization of BC@PST and BC@CMC Composites

**Figure 8:**
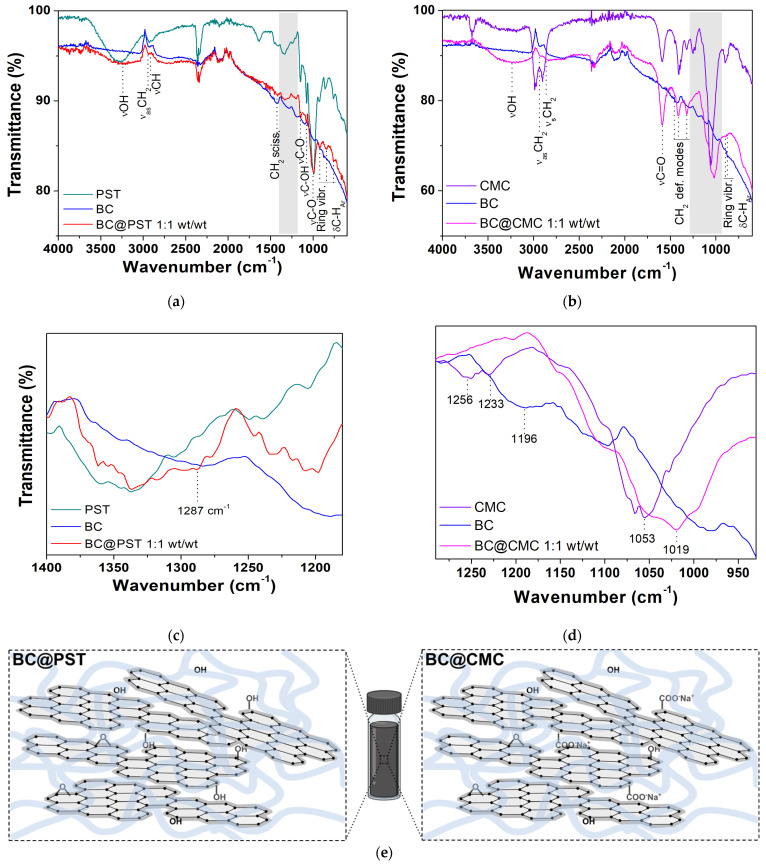
FT–IR ATR spectra: (**a**) full spectrum of potato starch (PST, dark cyan line), raw biochar (BC, blue line), and potato-starch-based composite in a 1:1 wt/wt ratio (BC@PST, red line); (**b**) full spectrum of sodium carboxymethylcellulose (CMC, violet line), raw biochar (BC, blue line), and CMC-based composite in a 1:1 wt/wt ratio (BC@CMC, magenta line); (**c**) 1400–1250 cm^−1^ wavenumber region taken from (**a**); (**d**) 1290–930 cm^−1^ wavenumber region taken from (**b**); (**e**) schematic representation of BC@polymer composite structure.

To further examine the chemical properties of the PST- and CMC-based composites and elucidate the interactions between counterparts, FT–IR spectra in ATR mode were recorded on dried composites. The FT–IR spectrum of pristine potato starch and CMC (taken as references) can be found in Figures S6 and S7, with complete band assignment. To reduce sampling bias and improve the poor signal-to-noise ratio in reflectance FT–IR measurements, an ad hoc sample was prepared for each composite at a 1:1 wt/wt ratio. The BC@PST and BC@CMC composites’ full FT–IR spectra (4000–600 cm^−1^) are presented in Figure 8. In both spectra (red and magenta lines in Figure 8a,b), a broad-stretching (ν) vibration band is observed in the 3030–3650 cm^−1^ region centered at 3238 cm^−1^ for BC@PST and at 3255 cm^−1^ for BC@CMC, which indicates the presence of hydroxyl (−OH) functional groups involved in hydrogen bonds. The absorption bands at ca. 2930 and 2840 cm^−1^ labeled as ν_as_(CH_2_) and ν(CH) are due to methylene −CH_2_ and −CH stretching vibrations, respectively, arising from both BC and polymers aliphatic chains, together with the −CH_2_ scissoring deformation modes in the 1420–1310 cm^−1^ wavenumber region, coupled with alcoholic O−H in-plane bending [78]. In the case of the CMC polymer matrix, the strong band at 1590 cm^−1^ pertains to hydrated carboxylic C=O stretching in the anion form of the CMC functional group. In the latter, the bands at 898 and 874 cm^−1^ are assigned to C−O−C vibrations of cellulose ring (coupled with aromatic C−H bending of biochar) [69]. In BC@PST, the stretching of the C−O−C and C−OH groups can be found at 1150, 1078, and 995 cm^−1^. In addition, the bands appeared at 926 (band type I, asymmetric deformation of the ring), 843 (band type II, deformation vibration of the −CH bond), and 753 cm^−1^ (band type III, characteristic for α-glycosidic bond), corresponding to the vibration of the carbohydrate ring [79,80], partially overlapping with the stretching vibration of the aromatic C−H of BC. A noticeable difference among pristine polymers, raw BC, and composite spectra can be seen in the fingerprint region (highlighted in gray in Figure 8a,b) and reported in Figure 8c,d. To establish a reasonable interaction occurring between biochar and polymer matrices, the calculated FT–IR spectra of the BC@PST and BC@CMC composite (obtained via 1:1 weighted sum of raw BC and pristine polymer spectra) were compared with the experimental one in Figure S8.

In the BC@PST spectrum (1400–1250 cm^−1^, Figure 8c), a broadening of the band centered at 1287 cm^−1^, which involved the C−O stretching of ethers vibrations, with no significant shift compared with the calculated one. However, in the experimental spectrum band due to ν(−OH) at 3238 cm^−1^, it is much less intense compared with a calculated physical mix of BC and PST (Figure S8), whereas that calculated profile linearly follows the experimental one in the 1400–1180 cm^−1^ region, making it possible to hypothesize that the above-mentioned broadening at 1287 cm^−1^ was due to the BC and PST absorption combination. Then, the wavenumber of hydroxyl stretching vibration shifted from 3290 cm^−1^ (pristine PST) to a lower wavenumber 3238 cm^−1^ in the composite, while the characteristic band is absent in raw BC (see Figure 1 for a better comparison). The blueshift in the ν(−OH) vibration is due to the formation of intermolecular hydrogen bonds [81]. Furthermore, the heat treatment (applied in the preparation of the composite) is known to reduce the intramolecular hydrogen bonds among starch molecules [82]. Heating starch at temperature ≥70 °C cause hydrogen bonds to break, with water molecules that combine with −OH groups of starch and tend to swell, beginning an irreversible thermal transition called gelatinization and condensation (due to amylose fractions), which reflects in a sol formation [71,83]. Upon cooling, the amylose molecules re-associate to form a network during retrogradation [84]. Thus, based on the (1) decrease in the ν(−OH) band intensity, (2) blueshift in the wavenumber of ν(−OH) in the FT–IR spectrum, and (3) PST gel-like network formation upon retrogradation, it is possible to ascribe the BC stabilization in the BC@PST composite to a combination of both hydrogen bonds and physical entrapment. Considering the sedimentation tests (Section 3.3.1) and ζ-potential results (Section 3.3.2), physical entrapment plays a major role in the stabilization, since a higher concentration of PST reduces BC sedimentation over time.

Conversely, in the BC@CMC composite spectrum (1290–930 cm^−1^ range, Figure 8d), the bands centered at 1256 and 1233 cm^−1^ related to the C−O stretching of the carboxylic group [69], typical of pristine CMC, are absent, as well as the band at 1196 cm^−1^ of raw BC. Moreover, a shift of 34 cm^−1^ occurs in the C−OH stretching vibration (from 1053 cm^−1^ in CMC to 1019 cm^−1^ in BC@CMC). The observed shift refers to the changes that occurred in the molecular structure of CMC [85]. The consistent broadening of this band may result in an interaction-driven combination of both C−O stretching vibrations of CMC and BC. Compared with the mere sum of absorptions, the experimental spectrum maintains its peculiar features: (a) the bandwidth of ν(−OH) centered at 3255 cm^−1^ and (b) the 34 cm^−1^ blueshift of ν(C−O) at 1019 cm^−1^, making it possible to hypothesize a strong hydrogen bond interaction involving C−O−C groups as H-acceptors and aliphatic C−O/C−OH as both H-donors/acceptors, thus stabilizing the composite structure to some extent, in agreement with similar composite of colloidal activated carbon stabilized by CMC matrix [12]. These results indicated no covalent bonds formation between polymers and BC but rather a combination of hydrogen bonds, hydrophobic forces, and physical entrapment, as roughly represented in Figure 8e.

### 3.4. Continuous Flow Column Distribution Tests on BC@Polymer Composites

Column distribution tests were carried out on the most promising BC composites in terms of stability over time to assess their properties as an injectable adsorption material. Therefore, the tests aimed to verify the actual possibility of distributing the suspension in a porous medium and to evaluate possible modifications to the composition of the suspension to optimize its fluid–dynamic behavior. The main qualities of the BC composites evaluated were (i) their ability to be transported throughout the whole column without generating clogging and (ii) being retained by the porous media during subsequent water-flushing tests. In the following section, the continuous flow column distribution tests on BC@PST, BC@CMC with CMC at 20 g/L, and BC@CMC with CMC 10 g/L are presented.

#### 3.4.1. Continuous Flow Column Distribution Tests on BC@PST Composite

**Figure 9:**
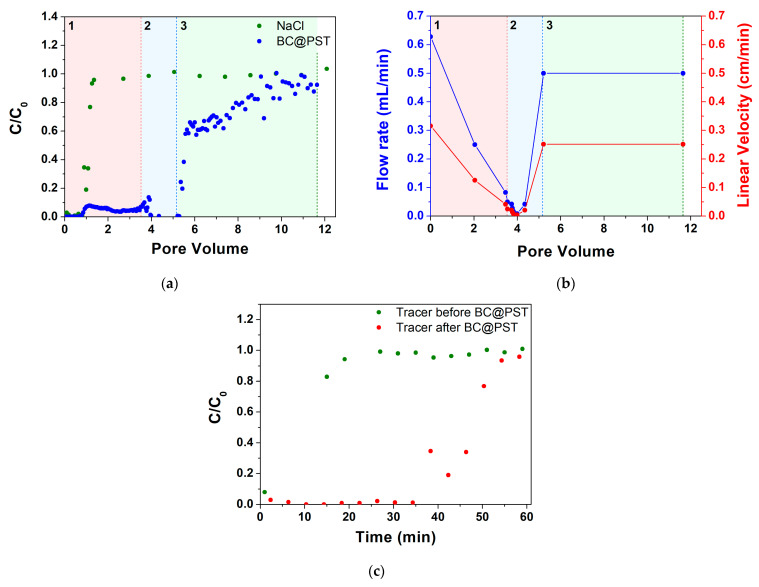
Tracer test on BC@PST: (**a**) step input tracer test with NaCl curve and BC@PST distribution test curve—C_0_ is the feed concentration, while C is the discharge concentration; (**b**) flow rate during the distribution test—section A (red), section B (blue), section C (green) are highlighted; (**c**) comparison between step input tracer test before and after BC@PST distribution test.

The first formulation that was deemed suitable for column distribution tests was BC@PST. On the column, packed as per paragraph 2.5, a tracer test was performed (Figure 9a), showing an effective volume (volume of empty pores) of 27.8 mL, an effective porosity (ε) of 0.41, and an initial hydraulic retention time (θ) of 44.3 min. The flow rate and linear velocity values in the 0–12 pore volume range are reported in Figure 9b.

Observing the trend of the breakthrough curves (Figure 9a), it can be divided into three sections in terms of pore volume (PV, the ratio between the volume of eluted pores/volume of empty pores): (1) PV 0–3.46, (2) PV 3.46–5.21, and (3) PV 5.21–11.64 range.

Section (1) highlights, in accordance with the tracer test, that the C/C_0_ of BC at the starting point is 0 and remains stable up to 0.78 PV, after which there is an increase in up to PV 1.13, where it stands at a value of 0.08. At this point there is a halving in C/C_0_ value to 0.04–0.05 up to PV 3.46, with a very stable trend. At the same time (between PV 0 and 3.46), a progressive decrease in the flow rate from 0.63 to 0.083 mL/min is registered (Figure 9b).

In the first part of section (2), between PV 3.46 and PV 3.99, there is a destabilization in the C/C_0_ value, varying between 0.01 and 0.14; while, at the same time, there is a sharp decrease in the flow rate from 0.083 down to 0.0083 mL/min (Figure 9b). Then, a high retention with C/C_0_ stable at 0.01 and a flow rate around 0.0083 to 0.042 mL/min occurs. Coherently, in the range PV 4.35–5.21, a flow rate increase from 0.042 to 0.5 mL/min was registered, which managed to remain at constant values for the rest of the test.

In section (3), a rapid increase in C/C_0_ from 0.01 to 0.58 in the range PV 5.28–5.57 can be observed. The unclogging was achieved by increasing the rpm of the pump for a couple of seconds. The rapid increase in C/C_0_ has a sharp drop in slope in the final section from PV 5.57 onwards, finally reaching an average value of 0.92 C/C_0_ at PV ca. 11. The residual mass in the column at the end of the test calculated using Equation (4) was 31.3 mg. Looking at the above results, a correlation between BC retention and workflow rate is clear, which is in agreement with various authors who find higher quantity of strained colloid for lower pore water velocity [36,86,87].

In order to assess the residual PV when loaded with BC@PST, a second test was performed with a NaCl tracer at the same flow rate as per the first test (Figure 9c). Due to the absence of points at the inflection of the test performed after loading with BC@PST, it is not possible to determine the exact θ value, which is, however, approximately a quarter of the initial one, thus giving us an ε of roughly 0.1 that justifies clogging issues. Due to the low mass retained in the column, the low maximum working concentration, and the difficulties in distribution due to clogging, it was subsequently decided to proceed with the study of other composites. In this case, the clogging is not due to mechanical filtration as there is no evidence of cake formation on the feed side of the column and is, therefore, inevitably attributable to physical straining.

#### 3.4.2. Continuous Flow Column Distribution Tests on BC@CMC 20 g·L^−1^ Composite

**Figure 10:**
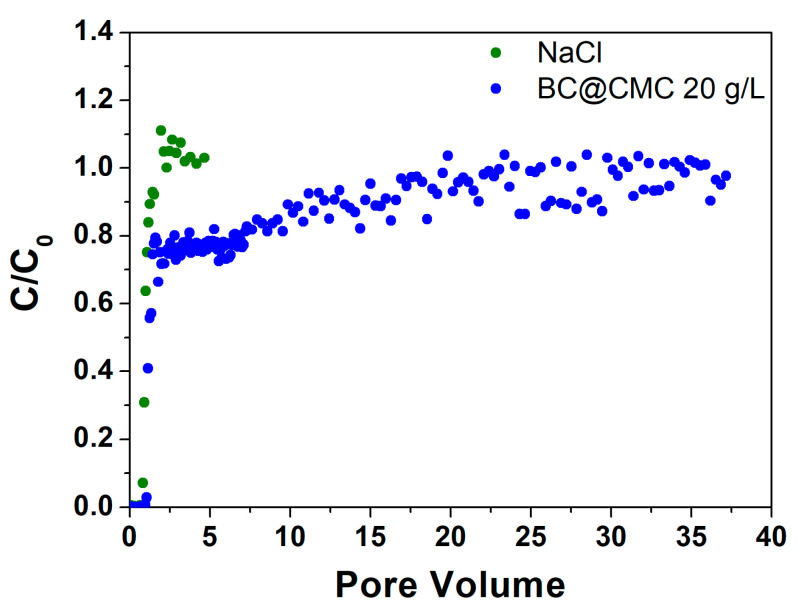
Step input tracer test with NaCl curve and BC@CMC with 20 g·L^−1^ distribution test curve.

The tracer test conducted on BC@CMC with CMC 20 g/L (Figure 10) showed a PV of 27.5 mL corresponding to an ε of 0.40 with a θ of 44.3 min. Similarly to the previous test, the BC in the effluent remains at 0 throughout the first PV, rises sharply to about 0.75 at PV 1.1, and remains constant until PV 6.4. At this point, a slow growth can be observed until it stabilizes around PV 30 at the end of the elution test, where an average C/C_0_ value of 0.98 is obtained between PV 30 and 37. At the end of the distribution test, a water rinse test was performed to remove the BC in the column not retained by the bed. However, after about 0.3 PV, the column became completely clogged, and it was impossible to continue the test. One hypothesis on clogging could be due to a sudden decrease in ionic strength following flushing. The retention of a colloid in a porous medium by straining is, in fact, strongly affected by the ionic strength; when the latter increases, the retention of the colloid increases [36,88]. Elution with deionized water causes an immediate zeroing of the ionic strength in the column, which had previously been at 81 mM (given by the CMC), generating an immediate release of a fraction of BC retained by attachment processes to clog the column by physical straining. In this case, it was possible to calculate the d_p_/d_c_ ratio, which stands at a value of 0.00361–0.00481, in line with theoretical values that guarantee physical straining [35,89], confirming the aforementioned filtration mechanism. In this case, due to the higher concentration achievable (C_0_), the residual mass in the column at the end of the test calculated was >128 mg, higher than the value previously obtained. From this test, it can certainly be deduced that CMC provides better properties than PST with respect to the transport of BC (as the distribution test was performed), without any variation in the flow rate. Therefore, BC@CMC did not give any evident clogging issues. However, it is evident that further optimization of the suspension composition is required to avoid blocking the column, which then prevents future adsorption tests.

#### 3.4.3. Continuous Flow Column Distribution Tests on BC@CMC 10 g·L^−1^ Composite

**Figure 11:**
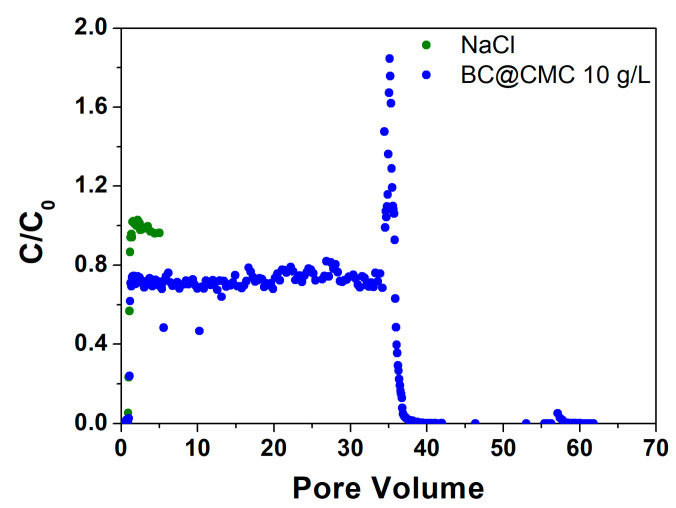
Step input tracer test with NaCl curve and BC@CMC 10 g·L^−1^ distribution test curve.

Based on the results obtained with BC@PST and BC@CMC, with CMC 20 g·L^−1^, the composite formulation was optimized by reducing the CMC concentration at 10 g·L^−1^. The related column distribution tests are presented in Figure 11. The reduction in the CMC concentration slightly reduces the stability of the BC, as can be observed from the sedimentation tests (Figure 6d in Section 3.3.1). However, it also reduces the hydrodynamic diameter of the particles that remain suspended (see Table S1), reducing the probability of clogging by physical straining following a massive release caused by ionic strength collapse. For this trial, the tracer test showed a PV of 29.8 mL, corresponding to an ε of 0.43 with a θ of 48.0 min. In agreement with data collected in previous tests, the concentration of BC begins to increase rapidly in the effluent shortly after the tracer breakthrough. Although, in this case, it reaches a C/C_0_ plateau of about 0.72 between PV 1.6 and 34.1 (where the flushing test is initiated), mobilizing significant amounts of BC with a C/C_0_ peak of 1.85. Thereafter, the concentration drops very rapidly and remains near zero for the duration of the flushing test of another 25 mL PV. Again, it is evident how the sudden lowering of the ionic strength leads to the mobilization of BC withheld by attachment, only due to a higher ionic strength (see Section 3.4.2) [77]. Instead of obtaining a clogging of the column, a strong momentary release is registered, evidenced by the C/C_0_ peak at 1.85. This result indicates that, in this case, the hydrodynamic diameter is small enough, (1122 ± 300) nm vs. (2887 ± 950) nm (see Table S1 for complete hydrodynamic diameter values), to not to clog the porous medium following massive release. A further interesting aspect of this test is the obtained plateau C/C_0_ of 0.72, significantly lower than the values of the previous tests of 0.92 and 0.98 for BC@PST and BC@CMC 20 g·L, respectively. This indicates a significantly more effective retention which does not lead to clogging. This could be due to a physical straining of a specific particle size fraction that is systematically retained by the porous medium, generating the steady state between PV 1.6 and 34.1 [34]. In this case, the residual mass in the column at the end of the test was higher than the one previously achieved, which was around 365 mg. It was possible to calculate the d_p_/d_c_ ratio to be 0.0014 and 0.0019, partially in line with the literature values that guarantee physical straining [35,78]. Indeed, the suitability of CMC as a dispersive matrix for carbonaceous materials was also reported for similar injectable colloidal activated carbon (CAC) stabilized by both CMC and humic acid (HA) [12]. In particular, it was further evidenced how the use of a polymer matrix improves the transport and distribution of the colloidal carbon (up to 10 g·L^−1^) in a fixed-bed column. However, it was demonstrated that physical straining dominated over attachment, as rinsing with deionized water removed only a fraction of the retained BC [77]. This test showed positive results concerning: (1) high amount of BC retained in the column, (2) efficient distribution of the suspension in the porous medium, and (3) constant flow rate throughout the transport and flushing test. Thus, the herein-studied BC@CMC (BC 1 g·L^−1^ and CMC 10 g·L^−1^) showed the most appropriate transport properties to be used as potential injectable permeable barrier for groundwater remediation.

## 4. Conclusions

In this work, biochars (BCs) waste obtained from the gasification of pine wood pellets (waste deriving from biomass energy production) at 950 °C were used as fillers in polymer matrices, to obtain BC@biopolymer composites for in situ groundwater remediation. The focus was on stability studies in water via UV–Vis and DLS and elucidating the role of polymers in stabilizing a biochar matrix. This information can advance the current state of the art in the optimization of the distribution and adsorption properties of organic contaminants in groundwater. Different biochar samples were studied, based on different sieving and grinding processes. Extensive characterizations were carried out on both BC and BC@polymer composites. Raw BCs showed a typical graphite-based structure with little to no residual functional groups, as evidenced by FTIR and UV–Visible spectroscopy. In water suspension, the grinding process resulted in a more intense population centered at around 200 nm with the formation of larger aggregates above 1000 nm with a sponge-like morphology of the surface and quasi-spherical pores. The micro-/mesoporous nature of BCs arose from BET and textural parameters analysis, with a reduction in the specific surface area of about 30% after the grinding process. Then, water-based polymer composites with chitosan (CS), alginate (ALG), potato starch (PST), and sodium carboxymethylcellulose (CMC) as polymer matrices (0.2–20.0 g/L concentration range) were synthesized via a simple blending approach on the selected biochar sample. According to pilot sedimentation tests, after 7 days, BC@PST (BC 0.3 g/L, PST 20.0 g/L + APG2 surfactant 1.0% *v*/*v*) and BC@CMC (BC 0.3 g/L, CMC 10.0 and 20.0 g/L) were the most stable suspensions, with a sedimentation percentage of (14 ± 1)%, (31 ± 2)%, (27 ± 2)% for BC@PST, BC@CMC 10 g/L, and BC@CMC 20.0 g/L, respectively. Further ζ-potential studies showed a decrease in the value of about 15% over a week for BC@PST, whereas for BC@CMC, it fluctuates around -55 mV at all time points. Although the BC@PST + APG2 formulations showed the lower sedimentation percentage compared to BC@CMC, the latter showed the highest colloidal stability over time. Enhanced stability of the mentioned composites was ascribed to a combination of both hydrogen bonds and physical entrapment, as studied by FTIR. Final composites formulations were optimized increasing the BC concentration up to 1.0 g/L.

Before the distribution tests on BC@PST and BC@CMC, the adsorption performances of the raw BCs were validated via an adsorption isotherm using trichloroethylene (TCE) as a model contaminant. The continuous flow column distribution tests on the composites resulted in a clogging of porous media in the case of BC@PST and BC@CMC 20.0 g/L (the latter after about 0.3 PV), probably due to a higher hydrodynamic diameter of composite particles in relation to the pore size of the media. Conversely, the BC@CMC 10.0 g/L composite showed an optimized distribution behavior (showing a plateau C/C_0_ of 0.72, significantly lower than the values of the previous tests of 0.92 and 0.98 for BC@PST and BC@CMC 20 g·L, respectively), with high retention (ca. 365 mg) and without column clogging. Thus, we were able to select CMC as the most suitable polymer for pine-wood-derived biochar stabilization in water (without any chemical modification or pre-treatment of the carbonaceous material), good transport, and retaining in breakthrough tests. The availability of such a stable biochar suspension opens to TCE in situ remediation as injectable permeable reactive barriers (IPRBs) overcoming traditional PRBs to address various groundwater pollution challenges. In situ injectable barriers allow for the achievement of the plume break, interrupting contaminants migration pathway.

## Figures and Tables

**Table 1:** Biochar (BC) samples notation studied in this work. All samples were obtained at 950 °C.

Sieving at 0.5 mm	Sieving at 64 µm and Grinding *
A	AP
B	BP

* P denotes additional manual grinding on sample.

**Table 2:** Conditions applied to obtain different BC@biopolymer composites in H_2_O_up_ depending on the polymer nature. These conditions were applied regardless of the BC (0.3–1.0 g·L^−1^) and polymer (0.2–20.0 g·L^−1^) concentrations used.

Type of Biopolymer	Temperature *	Reaction Time	Additive
Chitosan (CS)	Room temperature	2 h	-
Sodium alginate (ALG)	Room temperature	2 h	-
Potato starch (PST)	70 °C	1 h	APG2 1.0% *v*/*v*
Sodium carboxymethylcellulose (CMC)	Room temperature	2 h	-

* Temperature is supplied via immersion in an oil bath.

**Table 3:** Weight loss, BET specific surface area, total pore volume, and micro-pore volume.

BC Sample *	Weight Loss (%)	BET Specific Surface Area (m^2^·g^−1^)	Total Pore Volume (cm^3^·g^−1^)	Micro-Pore Volume (cm^3^·g^−1^)
A	6.59%	457 ± 5	0.511	0.098
AP	3.31%	293 ± 5	0.421	0.046
B	12.97%	523 ± 5	0.578	0.140
BP	3.37%	378 ± 5	0.474	0.087

* P denotes additional manual grinding on sample.

## Data Availability

The raw data supporting the conclusions of this article will be made available by the authors on request.

## Supplementary Materials

The following supporting information can be downloaded at: https://www.mdpi.com/article/10.3390/ma17163899/s1, Figure S1: UV-Vis spectra of BC samples dispersed in H2Oup: (a) sample A (sieving at 0.5 mm); (b) sample AP (sieving at 64 µm and grinding); (c) sample B (sieving at 0.5 mm); (d) sample BP (sieving at 64 µm and grinding); Figure S2: (–f) SEM images of raw pine wood biochar (AP sample) at different magnification drop-casted onto a silicon stub from an aqueous suspension. Accelerating voltage was 1.50 kV; (g) EDS of AP biochar sample; Figure S3: Pore size distribution for different raw biochar samples obtained at 950°C: (a,c) sample A, B (sieving at 0.5 mm); (b,d) sample AP, BP (sieving at 64 µm and grinding); Figure S4: DLS distribution in H2Oup of BC dispersion after 2 h aging: (a) sample A (sieving at 0.5 mm); (b) sample AP (sieving at 64 µm and grinding); (c) sample B (sieving at 0.5 mm); (d) sample BP (sieving at 64 µm and grinding). Inset: ζ-potential values; Figure S5: Time-dependent sedimentation of raw biochar (AP sample) at concentration 0.3 g/L mixed with different %v/v APG2 surfactant at t0, after 24 h, and after 7 days; Figure S6: (a) FTIR-ATR spectrum of commercial potato starch and (b) its assignment [90,91]; Figure S7: (a) FTIR-ATR spectrum of commercial sodium carboxymethylcellulose and (b) its assignment [90,91,92]; Figure S8: FTIR ATR spectra: (a) overlap between calculated spectrum of BC@PST (dotted line) and the spectrum obtained experimentally (continuous line); (b) overlap between calculated spectrum of BC@CMC (dotted line) and the spectrum obtained experimentally (continuous line); Table S1: Hydrodynamic parameters recorded on freshly prepared composite formulation and after 24 h and 7 days static aging at room temperature.
