# Supplementary material for: Biochar/Biopolymer Composites for Potential In Situ Groundwater Remediation
Source: Materials (Basel). 2024 Aug 6;17(16):3899. doi: 10.3390/ma17163899 (PMC11355651; doi:10.3390/ma17163899)
Supplement: Supplementary file 1 [file materials-17-03899-s001.zip › materials-3088662-supplementary.pdf]

Supporting Information

Biochar/Biopolymer Composites for Potential In Situ Groundwater Remediation

Marco Petrangeli Papini [1], Sara Cerra [1,*], Damiano Feriaud [1,*], Ida Pettiti [1], Laura Lorini [1] and Ilaria Fratoddi [1,2,3]

[1] Department of Chemistry, Sapienza University of Rome, Piazzale Aldo Moro 5, 00185 Rome, Italy; marco.petrangelipapini@uniroma1.it (M.P.P.); ida.pettiti@uniroma1.it (I.P.); laura.lorini@uniroma1.it (L.L.); ilaria.fratoddi@uniroma1.it (I.F.)

[2] Research Center for Applied Sciences to the Safeguard of Environment and Cultural Heritage (CIABC), Sapienza University of Rome, Piazzale Aldo Moro 5, 00185 Rome, Italy

[3] Research Center for Nanotechnology Applied to Engineering of Sapienza (CNIS), Sapienza University of Rome, Piazzale Aldo Moro 5, 00185 Rome, Italy

* Correspondence: sara.cerra@uniroma1.it (S.C.); damiano.feriaud@uniroma1.it (D.F.); Tel.: +39-06-4991-3352 (S.C.)

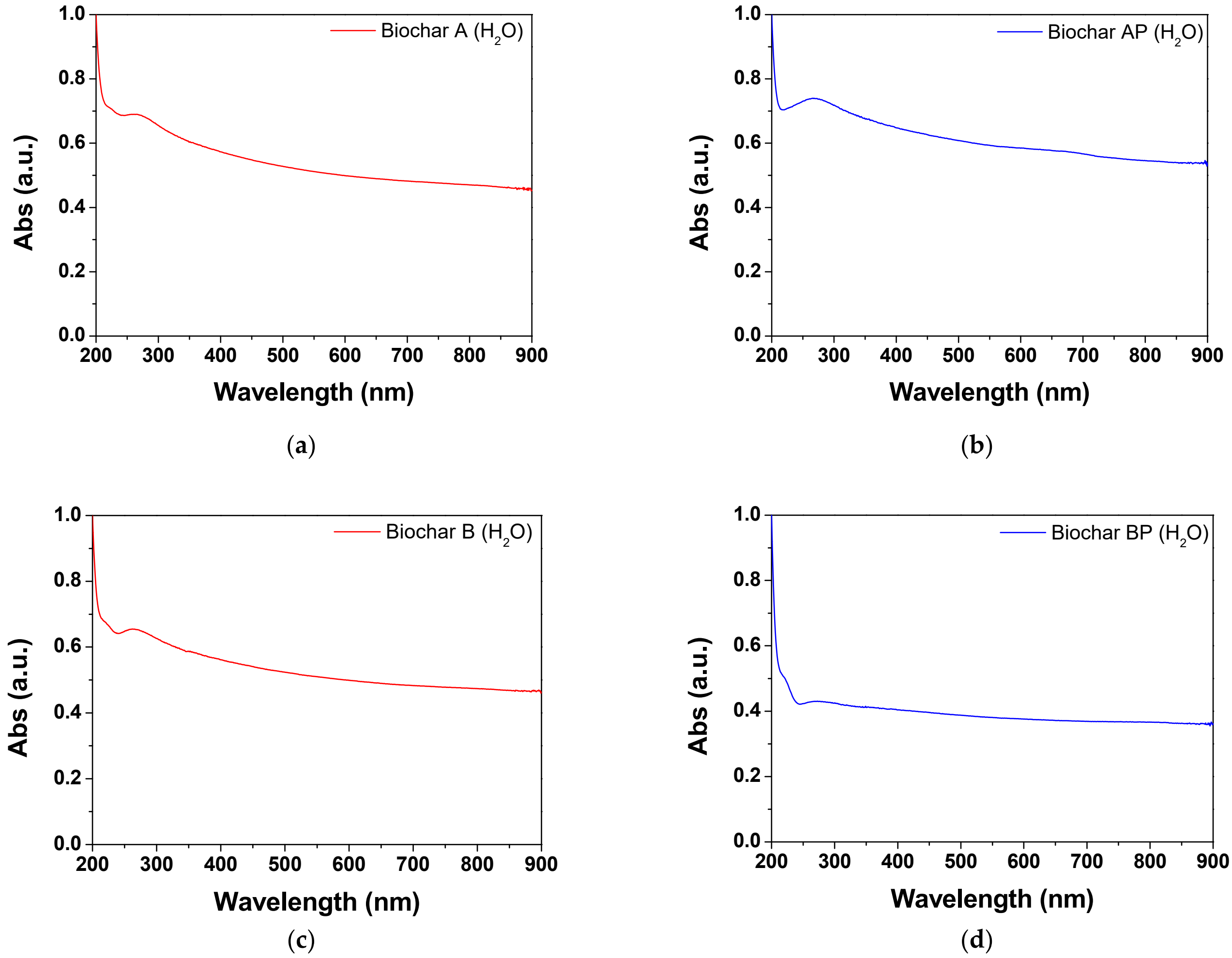

Figure S1. UV-Vis spectra of BC samples dispersed in H_2O_{up}: (**a**) sample A (sieving at 0.5 mm); (**b**) sample AP (sieving at 64 μm and grinding); (**c**) sample B (sieving at 0.5 mm); (**d**) sample BP (sieving at 64 μm and grinding).

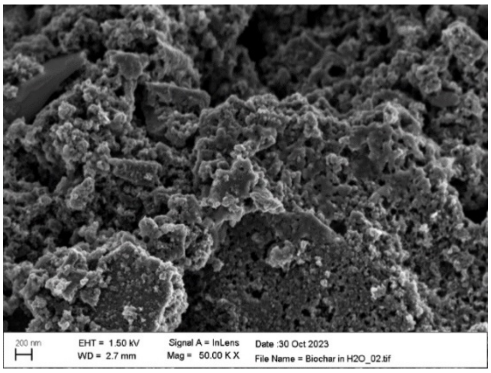

(**a**)

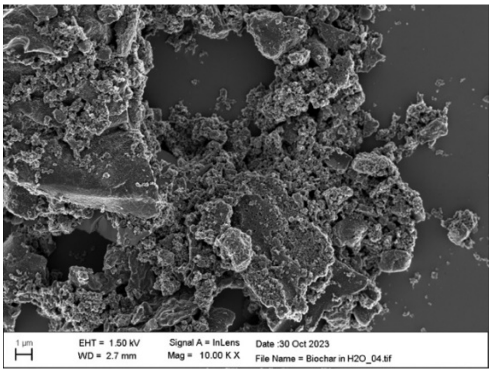

(**b**)

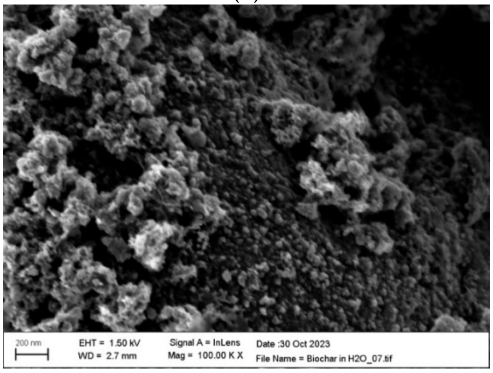

(c)

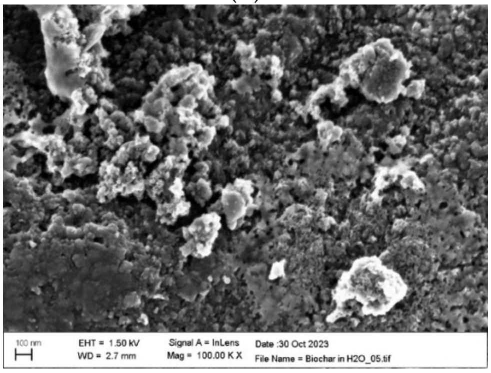

(d)

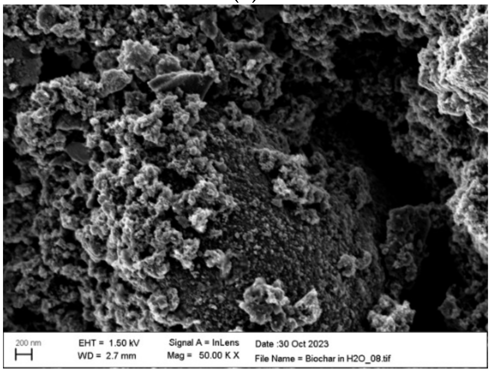

(e)

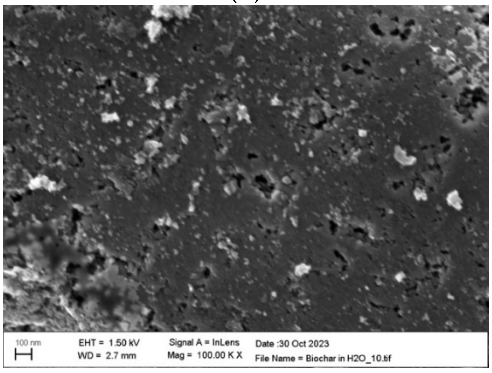

(f)

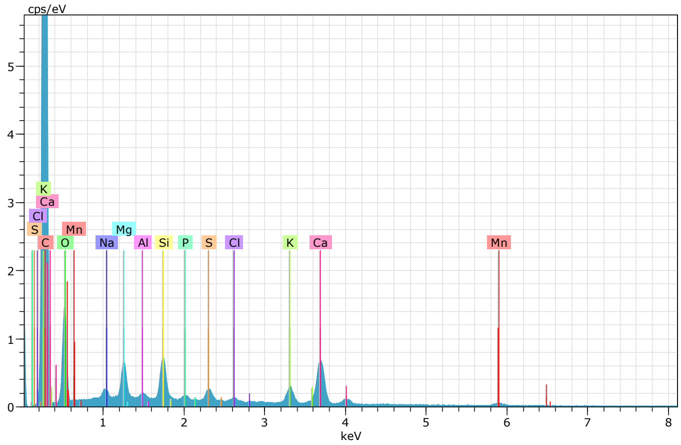

Element	Unn. [wt.%]	C norm. [wt.%]	C atom. [at.%]	2σ [wt.%]
Carbon	76	74	83	16
Oxygen	15	14	12	4
Sodium	<1	<1	<1	<1
Magnesium	1	1	<1	<1
Aluminium	<1	<1	<1	<1
Silicon	2	2	<1	<1
Sulfur	<1	<1	<1	<1
Phosphorous	<1	<1	<1	<1
Chlorine	<1	<1	<1	<1
Calcium	6	6	2	<1
Potassium	2	1	<1	<1
Manganese	1	1	<1	<1

(g)

Figure S2. (**a**–**f**) SEM images of raw pine wood biochar (AP sample) at different magnification drop-casted onto a silicon stub from an aqueous suspension. Accelerating voltage was 1.50 kV; (**g**) EDS of AP biochar sample.

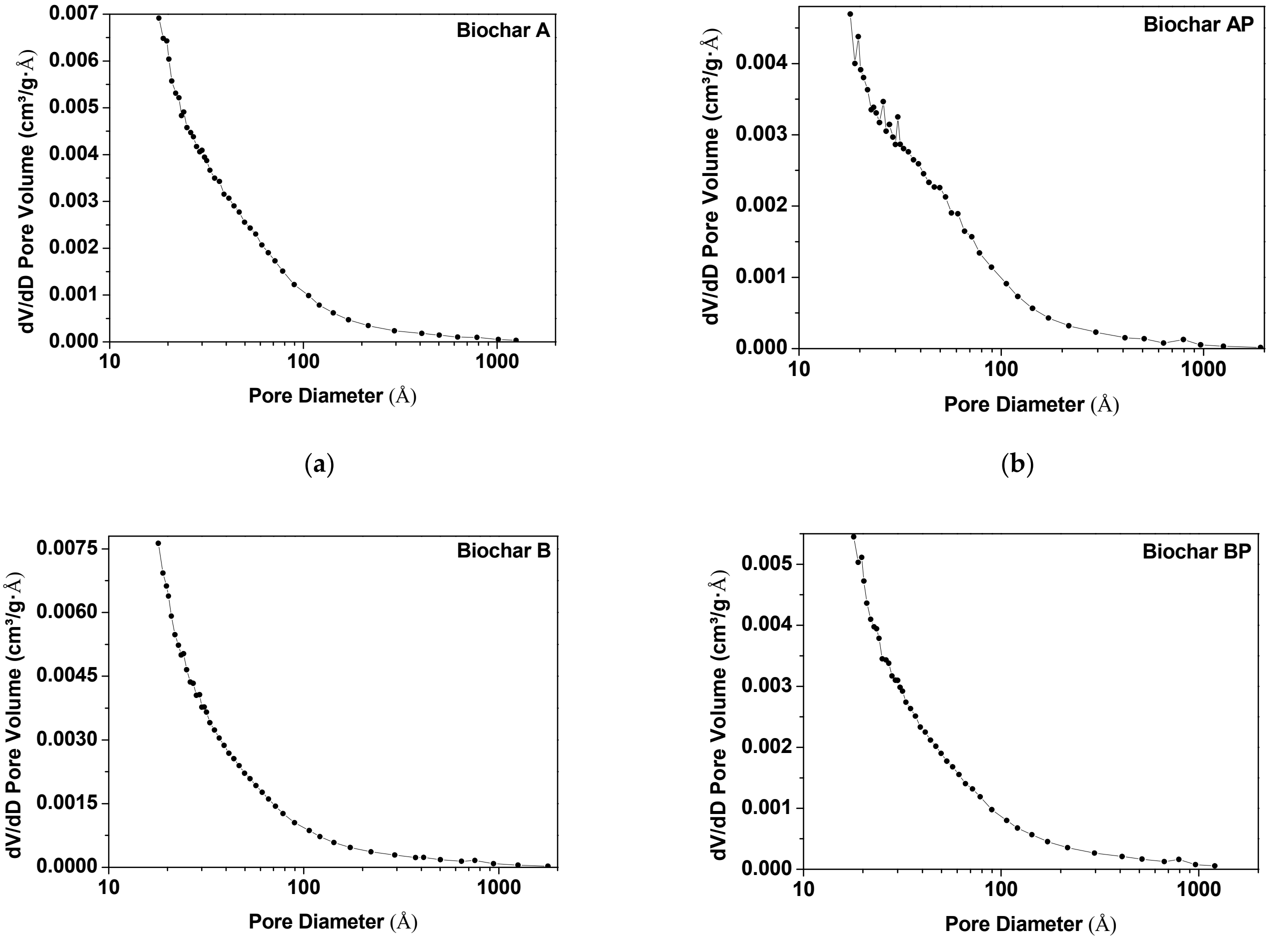

(c) (d)

Figure S3. Pore size distribution for different raw biochar samples obtained at 950°C: (**a,c**) sample A, B (sieving at 0.5 mm); (**b,d**) sample AP, BP (sieving at 64 μm and grinding).

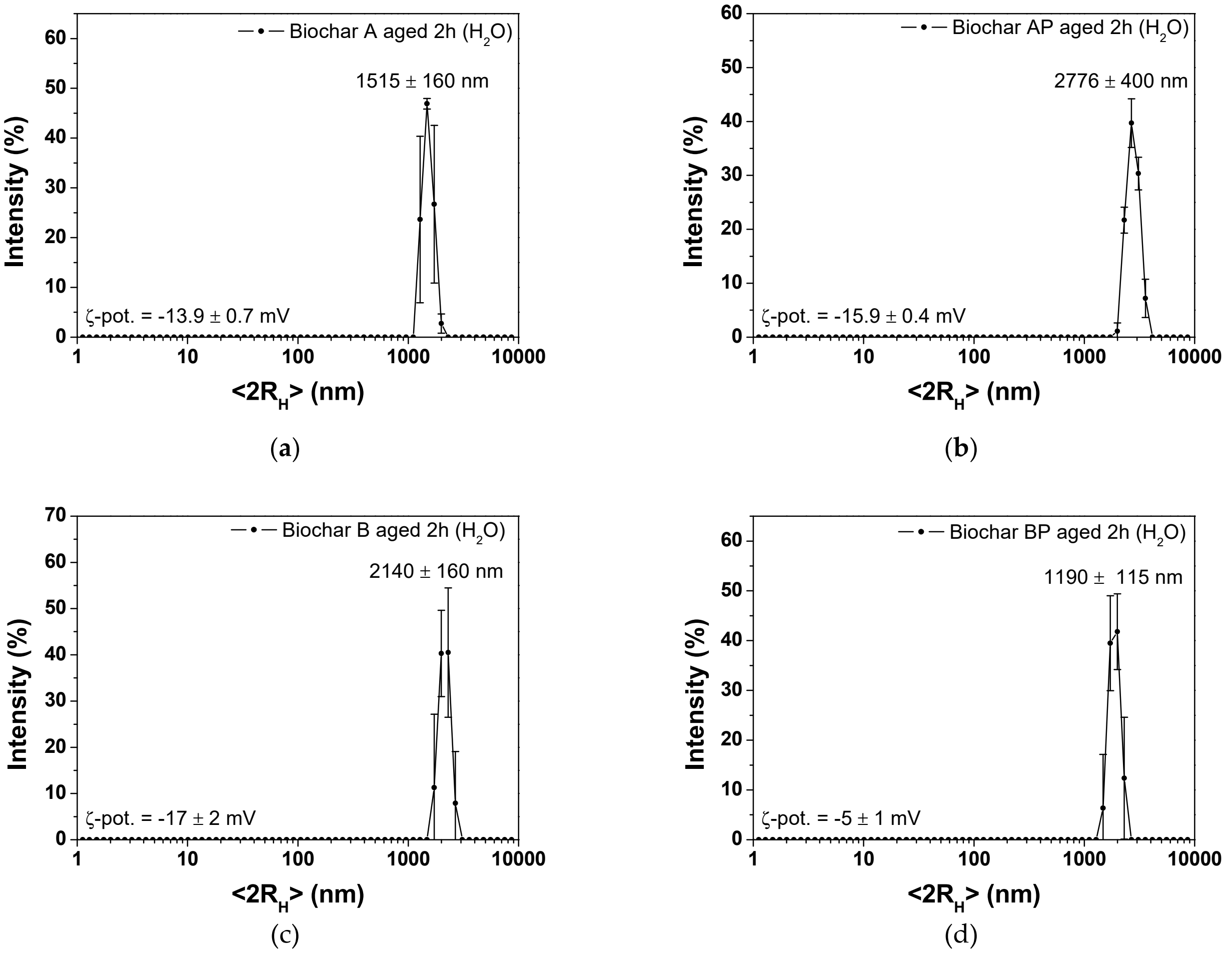

Figure S4. DLS distribution in H_2O_{up} of BC dispersion after 2 hours aging: (**a**) sample A (sieving at 0.5 mm); (**b**) sample AP (sieving at 64 μm and grinding); (**c**) sample B (sieving at 0.5 mm); (**d**) sample BP (sieving at 64 μm and grinding). Inset: ζ-potential values.

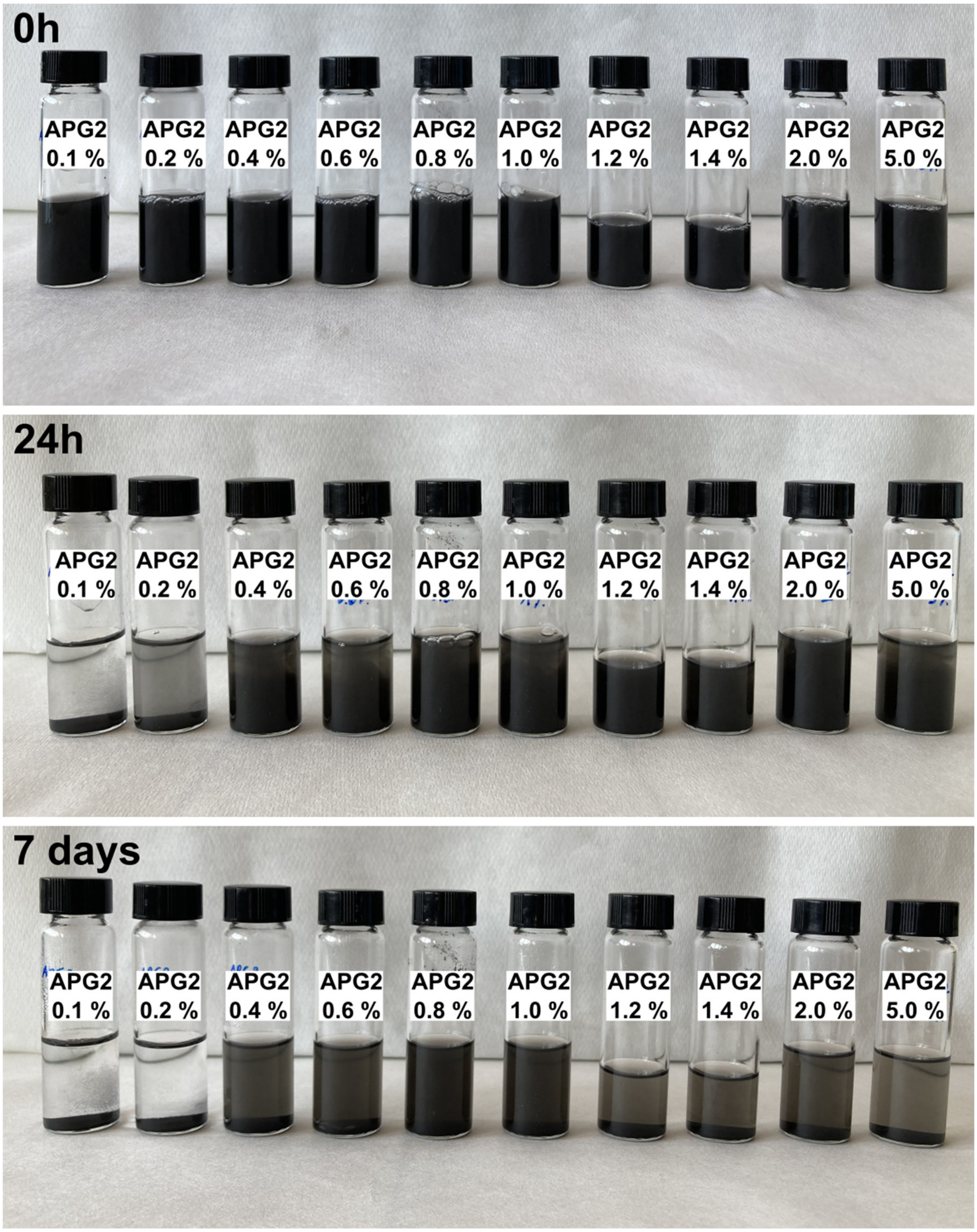

Figure S5. Time-dependent sedimentation of raw biochar (AP sample) at concentration 0.3 g/L mixed with different %v/v APG2 surfactant at t0, after 24 h, and after 7 days.

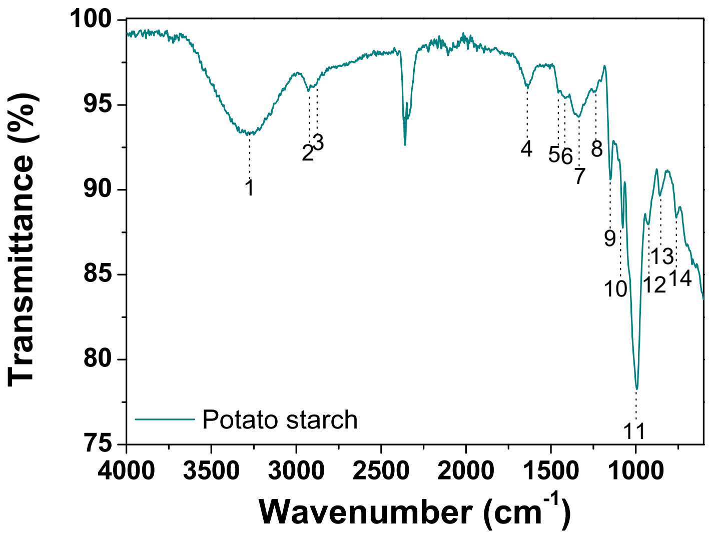

(a)

Band No.	Wavenumber (cm⁻¹)	Assignment [[1],[2],[3],[4]]
1	3290	O–H stretching
2	2930	CH_2 asymmetric stretching
3	2892	CH stretching
4	1642	–OH bending abs. water
5	1459	$-CH_2$ scissoring
6	1421	–OH in-plane bending + C–H wagging
7	1340	–OH in-plane bending + C–H wagging
8	1245	C–O–C stretching of ethers
10	1151	
11	1078	C–OH stretching
12	995	
13	929	Asymmetric deformation of the carbohydrate ring
14	856	Deformation vibration of the CH bond at the glycosidic carbon atom
15	763	Symmetric ring vibration

(b)

Figure S6. (**a**) FTIR-ATR spectrum of commercial potato starch and (**b**) its assignment [90,91].

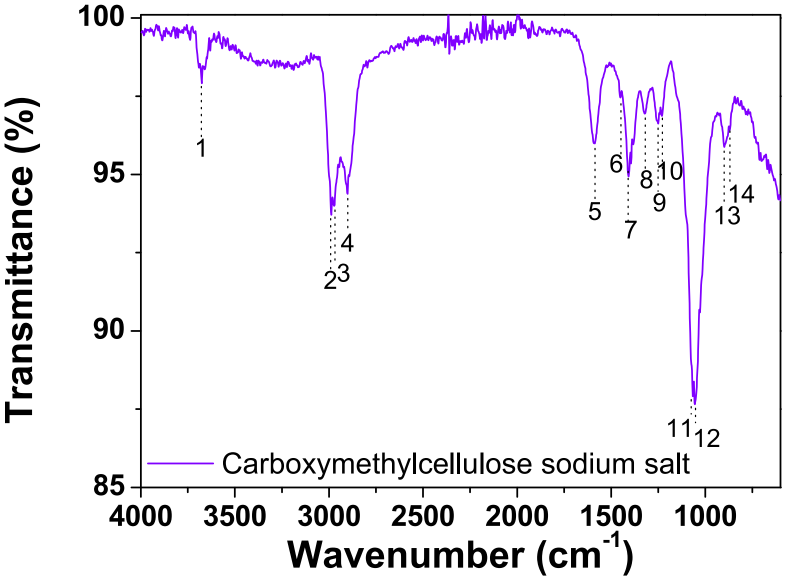

(a)

Band No.	Wavenumber (cm^{-1})	**Assignment [**Error! Bookmark not defined.**,**Error! Bookmark not defined.**,[5]]**
1	3676	O–H stretching
2	2988	CH_2 asymmetric stretching
3	2972	CH_2 symmetric stretching
4	2901	CH stretching
5	1592	C=O stretching
6	1452	CH_2 scissoring
7	1415	–OH in-plane bending + C–H wagging
8	1324	–OH in-plane bending + C–H wagging
9	1256	C–O stretching of C(O)-O^-
10	1233	
11	1070	C–OH stretching
12	1053	
13	897	Carbohydrate ring vibrations
14	874	

(b)

Figure S7. (**a**) FTIR-ATR spectrum of commercial sodium carboxymethylcellulose and (**b**) its assignment [90–92].

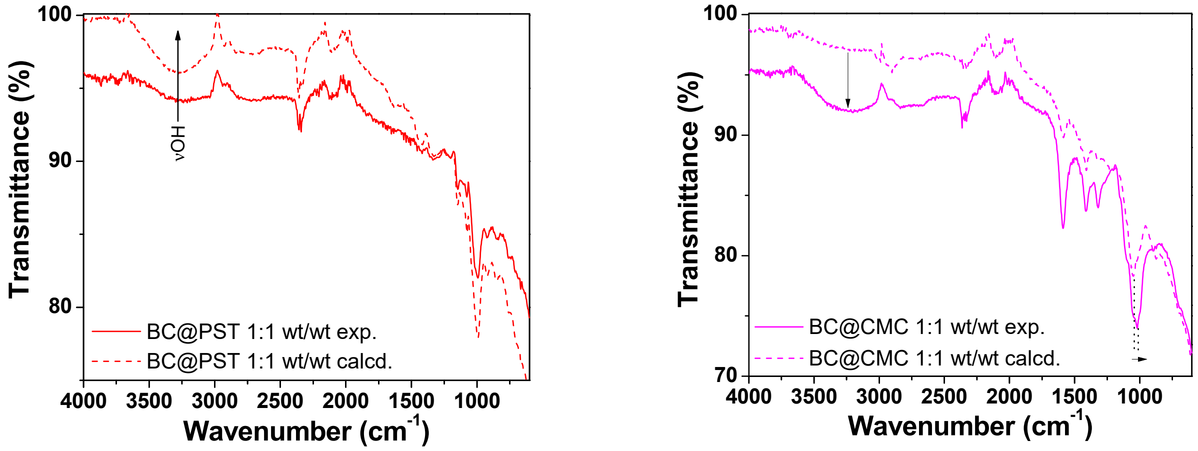

(**a**) (**b**)

Figure S8. FTIR ATR spectra: (**a**) overlap between calculated spectrum of BC@PST (dotted line) and the spectrum obtained experimentally (continuous line); (**b**) overlap between calculated spectrum of BC@CMC (dotted line) and the spectrum obtained experimentally (continuous line).

Table S1. Hydrodynamic parameters recorded on freshly prepared composite formulation and after 24 h and 7 days static aging at room temperature.

BC 0.3 g/L + Biopolymer		t=0		t=24h		t=7 days		
	Concentration (g/L)	<2R$_H$> (nm)	ζ-potential (mV)	<2R$_H$> (nm)	ζ-potential (mV)	<2R$_H$> (nm)	ζ-potential (mV)	pH*
CS	0.2	456 ± 260	+11.6 ± 0.9	706 ± 192	+28.1 ± 0.6	647 ± 174	+12 ± 1	6.63
	1	1091 ± 523	+58 ± 2	943 ± 207	+52.5 ± 0.1	750 ± 160	+48 ± 2	5.52
	5	952 ± 194	+62 ± 2	1088± 296	+66 ± 1	1050 ± 220	+59 ± 4	4.59
	7.5	1408 ± 312	+64 ± 2	1025 ± 264	+63 ± 1	1917 ± 1146	+62.4 ± 0.4	4.56
	10	1844 ± 508	+62 ± 3	2663 ± 473	+53 ± 3	4791 ± 722	+60 ± 3	4.46
	15	1333 ± 215	+63 ± 4	1260 ± 180	+63 ± 2	2028 ± 520	+62 ± 4	4.39
	20	1282 ± 219	+65 ± 6	5213 ± 469	+63 ± 3	4901 ± 676	+62 ± 3	4.39
ALG	0.2	543 ± 121	-47.4 ± 0.6	428 ± 278	-43.7 ± 0.6	479 ± 257	-51 ± 4	9.34
	1	576 ± 148	-64 ± 2	587 ± 254	-70 ± 5	475 ± 158	-63.2 ± 2	9.29
	5	1091 ± 255	-62 ± 1	868 ± 220	-64.4 ± 0.5	623 ± 82	-60 ± 3	9.05
	7.5	1073 ± 468	-59 ± 1	1023 ± 359	-63.9 ± 0.8	956 ± 256	-59 ± 1	8.77
	10	902 ± 287	-59.1 ± 0.2	1009 ± 246	-56 ± 2	1049 ± 216	-57.4 ± 0.7	8.47
	15	1078 ± 452	-58 ± 3	1037 ± 285	-59 ± 2	979 ± 176	-57 ± 4	7.93
	20	1481 ± 279	-42 ± 1	1041 ± 258	-60 ± 3	1259 ± 355	-57 ± 1	7.14
**PST+APG2 1%	0.2	338 ± 140	-32 ± 2	376 ± 182	-24 ± 2	430 ± 193	-30 ± 1	9.16
	1	465 ± 170	-30 ± 2	1140 ± 304	-12 ± 3	935 ± 215	-27 ± 2	9.26
	5	-	-19 ± 3	-	-11 ± 1	-	-14 ± 3	9.22
	7.5	-	-12 ± 3	-	-10 ± 1	-	-16 ± 2	9.09
	10	-	-15.6 ± 0.8	-	-8 ± 2	-	-14 ± 3	8.84
	15	-	-16 ± 2	-	-17 ± 3	-	-12 ± 2	8.72
	20	-	-16 ± 2	-	-10.7 ± 0.4	-	-10.7 ± 0.8	8.85
CMC	0.2	706 ± 176	-46.0 ± 0.9	934 ± 235	-24.4 ± 0.9	741 ± 184	-36.2 ± 0.9	9.03
	1	961 ± 239	-67 ± 4	770 ± 163	-67 ± 3	786 ± 194	-55.6 ± 0.7	9.27
	5	994 ± 364	-62 ± 2	1266 ± 380	-57.0 ± 0.8	952 ± 242	-56 ± 2	9.21
	7.5	1383 ± 620	-56 ± 2	1124 ± 324	-55.7 ± 0.7	1166 ± 606	-54 ± 4	9.18
	10	1109 ± 291	-54 ± 2	1221 ± 394	-54 ± 4	1122 ± 300	-52.7 ± 0.5	9.12
	15	1403 ± 395	-55 ± 1	1438 ± 330	-54 ± 2	1454 ± 453	-51 ± 1	8.82
	20	1321 ± 344	-54 ± 1	1419 ± 297	-51 ± 1	2887 ± 950	-49 ± 2	8.92

*Standard deviation on the value is ± 0.01.

**Hydrodynamic diameters not reported in the table were out of range for DLS analysis.

References

90. Abdullah, A.H.D.; Chalimah, S.; Primadona, I.; Hanantyo, M.H.G. Physical and chemical properties of corn, cassava, and potato starchs. *Conf. Ser.: Earth Environ. Sci.* **2018**, *160*, 012003. https://doi.org/10.1088/1755-1315/160/1/012003.
91. Morán; J. I.; Vázquez; A.; Cyras, V. P. Bio-nanocomposites based on derivatized potato starch and cellulose, preparation and characterization. *J. Mater Sci.* **2013**, *48*, 7196–7203. https://doi.org/10.1007/s10853-013-7536-x.
92. Shehap, A.M. Thermal and Spectroscopic Studies of Polyviny Alcohol/Sodium Carboxy Methyl Cellulose Blends. *Egypt. J. Solids* **2008**, *31*, 75–91. https://doi.org/10.21608/EJS.2008.148824.
